# The Increasing Role of Short-Term Sperm Storage and Cryopreservation in Conserving Threatened Amphibian Species

**DOI:** 10.3390/ani13132094

**Published:** 2023-06-24

**Authors:** Zara M. Anastas, Phillip G. Byrne, Justine K. O’Brien, Rebecca J. Hobbs, Rose Upton, Aimee J. Silla

**Affiliations:** 1 School of Earth, Atmospheric and Life Sciences, University of Wollongong, Wollongong, NSW 2522, Australia; pbyrne@uow.edu.au (P.G.B.); asilla@uow.edu.au (A.J.S.); 2 Taronga Institute of Science and Learning, Taronga Conservation Society Australia, Mosman, NSW 2088, Australia; jobrien@zoo.nsw.gov.au (J.K.O.); rhobbs@zoo.nsw.gov.au (R.J.H.); 3 Conservation Science Research Group, School of Environmental and Life Sciences, University of Newcastle, Callaghan, NSW 2308, Australia; rose.upton@uon.edu.au

**Keywords:** amphibian, frog, conservation, assisted reproductive technologies, biobanking, cryopreservation, sperm, spermiation, IVF

## Abstract

**Simple Summary:**

Integrating reproductive technologies into amphibian conservation breeding programs can enhance the propagation and the genetic management of threatened species. Advances in cold storage and cryopreservation of amphibian sperm, coupled with assisted fertilisation technologies, are valuable tools for long-term genetic management and are being applied to an increasing number of threatened species globally. This review discusses the role of sperm storage in amphibian conservation, presents the state of current technologies for the short-term cold storage and long-term cryopreservation of amphibian sperm, and discusses the generation of cryo-derived offspring.

**Abstract:**

Multidisciplinary approaches to conserve threatened species are required to curb biodiversity loss. Globally, amphibians are facing the most severe declines of any vertebrate class. In response, conservation breeding programs have been established in a growing number of amphibian species as a safeguard against further extinction. One of the main challenges to the long-term success of conservation breeding programs is the maintenance of genetic diversity, which, if lost, poses threats to the viability and adaptive potential of at-risk populations. Integrating reproductive technologies into conservation breeding programs can greatly assist genetic management and facilitate genetic exchange between captive and wild populations, as well as reinvigorate genetic diversity from expired genotypes. The generation of offspring produced via assisted fertilisation using frozen–thawed sperm has been achieved in a small but growing number of amphibian species and is poised to be a valuable tool for the genetic management of many more threatened species globally. This review discusses the role of sperm storage in amphibian conservation, presents the state of current technologies for the short-term cold storage and cryopreservation of amphibian sperm, and discusses the generation of cryo-derived offspring.

## 1. Introduction

Globally, we are witnessing unprecedented losses to biodiversity, which threatens ecological processes and the functioning of ecosystems. Though all vertebrate classes are facing biodiversity loss, amphibians are exhibiting the most severe declines of any vertebrate class, with an estimated 41% of all species threatened with extinction [1]. These declines are caused by multiple factors, including habitat destruction and fragmentation, invasive species, over-exploitation, environmental toxins, disease, and global climate change [2,3,4]. The largest and most intractable driver for global amphibian extinctions is emerging infectious diseases [4,5,6]. Of these diseases, chytridiomycosis, caused by the fungal pathogen *Batrachochytrium dendrobatidis* (*Bd*), is causing severe declines on a global scale and threatens populations within both pristine and altered habitats [7,8,9]. Despite considerable research attention into the mitigation of *Bd*, effective strategies to control the disease in the wild remain elusive [4]. Considering the complexity of the multiple threats to biodiversity that amphibians are facing, including the potential for synergistic effects, in situ conservation efforts alone are often insufficient for species recovery [4]. Subsequently, conservation practitioners are being encouraged to adopt integrated, multifaceted approaches to amphibian conservation, including the application of both ex situ and in situ strategies [10,11,12]. Reproductive technologies (RTs, also known as assisted reproductive technologies (ARTs)) offer a suite of tools to improve the propagation and genetic management of threatened species, including technologies to reinvigorate genetic diversity and facilitate genetic exchange between living captive and wild populations, as well as from expired generations [10,13,14]. While the development of reproductive technologies for amphibian conservation has lagged behind those of other vertebrate taxa, there has been increasing research attention in this field, summarised in several recent review articles [15,16,17,18,19,20,21,22,23]. Taken together with the present review, the information summarised aims to provide a platform for the further expansion of knowledge and increased application of amphibian reproductive technologies for threatened species recovery.

Herein, we review the role of reproductive technologies in amphibian conservation, focusing on the current state of protocols for the short-term storage and cryopreservation of amphibian sperm. We review factors affecting the viability and longevity of sperm during short-term cold storage and the potential use of antibiotics and antioxidants. Next, we discuss the protocols developed for amphibian sperm cryopreservation and the generation of offspring from frozen–thawed sperm. We draw attention to the need to further investigate the effects of sperm storage protocols on the viability of generated offspring; this knowledge will be important to direct future protocol development and encourage the routine use of these technologies in captive breeding programs. Throughout this review, we identify potential avenues for research which could advance the application of sperm storage for the conservation of threatened amphibians.

### Amphibian Conservation Breeding Programs and the Value of Integrating Reproductive Technologies (RTs)

Conservation breeding programs (CBPs) aim to assist threatened species recovery in a number of ways, including maintaining genetically representative captive assurance colonies, generating offspring for release to bolster wild populations (through population augmentation, translocations, and reintroductions), providing individuals for scientific research (including the development of RTs), acting as ambassadors for conservation education, and generating additional financial support for conservation [24,25]. Following the publication of the first Amphibian Conservation Action Plan (ACAP) in 2007, there was a substantial increase in the number of amphibian CBPs, with programs established for upwards of 75 amphibian species globally [26]. Unfortunately, the number of CBPs established is only a small fraction of the well over 2500 amphibian species currently threatened with extinction [1]. Established programs have reported evidence of successful captive breeding and the formation of self-sustaining breeding populations in the wild post-release in a proportion of the programs recently assessed (S2 Supporting Information in [26]). Despite such celebrated successes, many programs continue to face a suite of challenges, including difficulties in initiating breeding behaviour in captive settings, lack of genetic diversity and lack of resources [27]. The integration of RTs into amphibian CBPs has the potential to aid in overcoming some of these common challenges and enhance long-term program success [16,19].

Reproductive technologies include a suite of techniques aimed at understanding and enhancing reproductive outcomes and the genetic management of threatened species [10]. RTs include but are not limited to: monitoring reproductive physiology, hormone therapies to induce spawning in pairs and gamete release (spermiation or ovulation) in individuals, short-term cold storage of gametes, long-term cryopreservation and biobanking of sperm, tissue or cell lines, and assisted fertilisation (AF, also referred to as artificial fertilisation, or in vitro fertilisation) [10]. When integrated into existing CBPs, RTs have the potential to enhance reproductive output, improve genetic management, and establish repositories of living genetic resources [10]. In particular, the ability to improve genetic management can help to address long-term goals of CBPs, where the preservation of representative genetic diversity is of paramount importance [10].

In amphibians, AF has been conducted in a growing number of threatened and near threatened species, including the Albanian water frog (*Pelophylax shqipericus*) [28], Axolotl (*Ambystoma mexicanum*) [29], Balkan water frog (*Pelophylax kurtmuelleri*) [30], brown toadlet (*Pseudophryne bibronii*) [31,32], dusky gopher frog (*Lithobates sevosa*) [33,34,35], green and golden bell frog (*Litoria aurea*) [36], hellbender (*Cryptobranchus alleganiensis*) [37], Puerto Rican crested toad (*Peltophryne lemur*) [38], Southern corroboree frog (*Pseudophryne corroboree*) [39], and Wyoming toad (*Anaxyrus baxteri*) [40].

Effective genetic management is essential for threatened species recovery, largely because both ex situ and in situ populations are often small and genetically isolated, which can lead to a loss of genetic diversity (through genetic drift and directional selection) and in turn a loss of fitness (inbreeding depression) [41,42]. These issues compound over generations, and can heighten a species’ risk of extinction, particularly when coupled with a loss of adaptive potential [41,42,43]. Assisted fertilisation is a valuable tool for the genetic management of threatened species, allowing heightened control over the genetic history of offspring produced, including benefits such as allowing the gametes of behaviourally incompatible pairs to be combined [44], or maximizing the reproductive output of genetically valuable individuals [19]. Assisted fertilisation can also be used to conduct quantitative genetic studies that (i) test for genetic compatibility between animals within CPBs; (ii) test for genetic compatibility of populations where population augmentation is proposed; and (iii) explore the potential for assisted gene flow to improve the adaptive potential of wild populations [13]. Once AF has been used to test for the compatibility and combining ability of populations [13], these techniques can then be used to facilitate genetic exchange between captive and wild populations, which is an extremely powerful tool for the long-tern genetic management of threatened species [14]. Additionally, when AF is conducted using frozen–thawed sperm, the reproductive lifespan of individuals can be extended, and the genetic diversity of populations can be reinvigorated from expired genotypes decades into the future.

## 2. Gamete Storage and Its Use in Conservation

In order to conduct AF in externally fertilising amphibians (over 90% of species of this taxon fertilise externally), viable gametes need to be obtained from males and females. The asynchronous release of gametes following hormone administration is relatively common in amphibians, with the timing of gamete release more variable, and generally delayed, in females compared to males [19]. Once released, the effective storage of gametes prior to AF is therefore valuable in order to maintain fertilisation capacity [45]. Furthermore, the storage of gametes facilitates the movement of genetic material between conservation breeding program facilities. In turn, this can improve the genetic diversity and quality of subsequent offspring, and minimises the need to transport live animals, which would present associated welfare and disease transfer considerations [19]. Storage can be either short-term via refrigeration at low temperatures (0–10 °C) or long-term via cryopreservation (ultra-low temperatures).

The viability and fertilisation capacity of both sperm and oocytes (unfertilised eggs) decreases over time after release, with oocyte viability decreasing more rapidly than sperm in amphibians [19]. For example, cane toad (*Rhinella marina*) oocyte fertilisation capacity rapidly decreases from above 80% to below 20% following ten hours of storage at optimal temperature, whereas sperm of this species retain > 50% motility after seven days of storage [46]. Similarly, spotted grass frog (*Limnodynastes tasmaniensis*) oocyte fertilisation capacity drops from above 70% to below 50% after four hours of storage at optimal storage osmolality [47]. In some species, oocyte viability may decline even more rapidly, with Gunther’s toadlet (*Pseudophryne guentheri*) oocytes decreasing from above 90% fertilisation capacity to 65% after just 30 min of storage, while sperm retained > 50% motility after six days of storage [48]. At most, amphibian oocytes maintain fertilisation capacity for a maximum of a few hours, compared to sperm maintaining motility for days to weeks [45]. Of note, controlled amphibian AF studies investigating the impact of prolonged chilled storage on fertilisation capacity are currently lacking. Regardless, due to the rapid decline in oocyte fertilisation capacity after release, research efforts have preferentially been focused on developing and refining short-term cold storage protocols for sperm to provide greater flexibility and efficiency [49].

In addition to the benefits of short-term cold storage of amphibian gametes, cryopreservation can improve conservation breeding programs by allowing living genetic material to be kept for decades or more in long-term repositories. Cryopreserved cells and tissues can contribute to the generation of genome resource banks (GRBs, also referred to as biobanks, cryobanks, germplasm repositories, or frozen biorepositories) to aid amphibian conservation, which has gained momentum over the last decade [20]. These ‘biobanks’ not only create a repository of genetic material, but thawed gametes can be used strategically for genetic rescue in real-time by facilitating the exchange of genetic material between captive and wild populations, as well as living and expired genotypes, to maximise genetic diversity [13,14,16]. Of note, modelling studies have reported that the inclusion of cryopreservation into amphibian CBPs can both reduce monetary costs and enhance retention of genetic diversity [50,51].

In amphibian gamete cryopreservation, research attention has similarly been focused on sperm storage over oocyte storage, because attempts at cryopreserving oocytes or embryos have been unsuccessful [52]. Amphibian oocytes are comparatively large in size and yolk volume, and this, alongside other yolk characteristics, inhibits the penetration of cryoprotectants into the cell, which, in turn, leads to incomplete cell dehydration and the formation of lethal intracellular ice [52]. Despite this, there is growing promise that amphibian maternal haploid (oocytes) or embryonic diploid (embryos) genomes could be successfully cryopreserved in the future via technologies such as vitrification and thawing by rapid laser-warming, at least in species with oocytes/embryos less than 2 mm in diameter [52]. Nevertheless, this presents an interesting avenue for further research, to attempt to overcome past barriers to preserving maternal haploid and embryonic diploid genomes in suitable species.

## 3. Methods of Obtaining Sperm

In amphibians, sperm can either be obtained via the excision of the testes following euthanasia or following hormone therapies to induce spermiation in live males. Historically, testicular sperm was the focus of most amphibian sperm storage studies, leading to successful protocol development for both short-term cold storage and long-term cryopreservation of sperm from a variety of species (e.g. [36,46,53,54,55,56,57,58,59,60,61,62,63]). Whilst the process of amphibian testes removal is lethal, the collection of sperm following hormone administration avoids the sacrifice of reproductively mature males and the hormones administered exert only a temporary effect on male physiology. For this reason, it is the preferred method for sperm collection of endangered species as it allows genetically valuable males to continue contributing to CBP gene pools for generations to come [17]. It is important to note, however, that testes macerates still have a role in improving CBPs, whereby in the case of unexpected animal death or euthanasia, testes should be immediately extracted and stored to preserve this valuable genetic material.

The administration of reproductive hormones to induce spermiation has been achieved in an increasing number of amphibian species spanning a variety of families [45]. The main hormones used for this purpose are purified human chorionic gonadotropin (hCG) and synthetic gonadotropin-releasing hormone (GnRHa). Recently, there has also been interest in the administration of a combination of GnRHa with a dopamine antagonist. When developing hormone therapies, it is necessary to determine the optimal type, dose and administration frequency on a species-specific basis because male responses to hormone administration show interspecific variation [45]. Despite this variation, a number of studies exist which can be used as a starting point to inform the application of hormone therapies to novel species. These studies have been comprehensively reviewed recently and the reader is directed elsewhere for further information on developing amphibian spermiation protocols [18,19,45,64,65,66].

## 4. Protocols for the Short-Term Storage of Amphibian Sperm

In many species, the main determinants of sperm fertilisation capacity are the percentage of motile sperm (the proportion of sperm swimming), motility longevity, sperm viability (the proportion of sperm with an intact plasma membrane), and sperm velocity (sperm swimming speed), as these parameters determine the ability of a sperm cell to both reach and penetrate an egg [67]. In following, sperm storage protocols that maximise these parameters are imperative to improving subsequent AF success.

### 4.1. Storage Medium Osmolality and Temperature

Whilst several abiotic factors can influence sperm performance during short-term sperm storage, medium osmolality (sum of all dissolved solutes in solution) and temperature are two of the main determinants of sperm longevity during storage in fish and amphibians [64,68].

In externally fertilising fish and amphibians, the isotonic osmolality of the seminal plasma surrounding sperm cells in the testes keeps sperm immotile and inactive prior to ejaculation [69]. The rapid change in osmolality of the fertilisation medium initiates sperm motility post-ejaculation [68]. In externally fertilising species that reproduce in freshwater, the sudden decrease in osmolality experienced by ejaculated sperm cells initiates motility activation [68,69]. In externally fertilising amphibians, this hypotonic shock promotes an increase in intracellular adenosine monophosphate that, in turn, triggers protein phosphorylation cascades which are responsible for activating sperm motility [70]. Sperm activation leads to rapid depletion of energy reserves [71]. As such, limiting sperm activation by storing sperm in immobilising suspensions can promote sperm longevity. Accordingly, the sperm of externally fertilising freshwater taxa are generally stored in mediums with relatively high osmolality that are isotonic to the testes [72].

In amphibians, the osmolality of seminal plasma is isotonic to that of the blood plasma (~240 mOsmol/kg) [73]; therefore, amphibian sperm is generally stored in media of similar osmolality. It follows that many studies in short-term sperm storage use a simplified amphibian Ringer (SAR) solution, made up to an osmolality of around 200 mOsmol/kg [54,63,74]. Overall, protocols for the short-term storage of amphibian sperm generally involve storing sperm suspensions in media of high osmolality (relative to freshwater or amphibian urine) to keep them inactive throughout storage, before exposing them to solutions of lower osmolality to initiate motility activation for use in AF [75].

Like osmolality, the importance of optimal storage temperature is well established in externally fertilising taxa. Temperatures ranging from 0 to 5 °C are most commonly used for the short-term storage of amphibian sperm, and this taxon has a high tolerance to cold shock [49,72]. Low temperatures aid in reducing the metabolic rate and energy consumption of sperm as well as reducing bacterial growth [71,76,77]. The sperm of several toad species (*Anaxyrus americanus*, *A. baxteri*, *A. fowleri*, and *A. boreas*) have been reported to lose motility significantly less rapidly when immediately placed in cold storage at 4 °C compared to storage at room temperature [49]. Furthermore, a study in the Booroolong frog (*Litoria booroolongensis*) determined that sperm motility was highest when stored for 12 days at 5 °C, compared with 0, 10 and 20 °C temperatures [54]. Overall, the longevity of amphibian sperm in short-term cold storage at 0–5 °C has been reported in several species [45]. Silla and Langhorne [45] highlight the significant difference in the longevity of sperm suspensions obtained via testes maceration or as spermatophores (up to 4 weeks) compared with spermic urine (typically 3–4 days). It was suggested that several factors may contribute to the comparatively lower longevity of stored spermic urine, including lower suspension osmolality, lower sperm concentration, and higher bacterial abundance present within spermic urine [45]. Bacterial contamination of sperm suspensions and the use of antibiotics in cold storage are discussed below (Section 4.3).

### 4.2. Oxygenation

Sufficient gas exchange between stored sperm and atmospheric oxygen is considered necessary to avoid anoxia [72]. In fish, flushing sperm suspensions with air or oxygen has been shown to improve the longevity of sperm during short-term cold storage in a range of species [72,78]. In the Booroolong frog (*L. booroolongensis*), sperm suspensions that were aerated with pure oxygen or atmospheric oxygen every three days had significantly higher sperm motility over 21 days of storage [54]. Similarly, aeration significantly improved sperm motility in the first 24 hours of storage in Fowler’s toad (*A. fowleri*) [79]. Aeration of sperm suspensions can be achieved via automated aquarium pumps or by manual exposure and physical agitation of the suspension [19,79]. Whilst Browne et al. [46] found no effects of aeration on cane toad sperm, they only exposed samples to air every five days and did not agitate samples. Sedimentation, which can lead to anoxia, is minimised by physical shaking or agitation, and this approach has been successfully adopted by other studies [19,79,80]. To further improve sperm longevity, future research into the optimal aeration frequency and gas composition (air or pure oxygen) may prove beneficial. In addition, whilst it has been widely studied in fish, the significant positive effects of aeration on sperm storage has only been reported in two anuran species [54,79] and is yet to be investigated in any urodele species and/or amphibian species with internal fertilisation.

### 4.3. Bacterial Contamination and the Use of Antibiotics

Another factor that can affect sperm longevity in short-term storage is bacterial contamination. Bacterial contamination can negatively affect sperm in several ways in a range of taxa, including inducing morphological damage, reduced functionality, and premature sperm capacitation in mammals [81,82,83,84,85,86]; decreased motility, increased DNA fragmentation, and increased oxidative stress in birds [87,88]; and reduced longevity in an aquatic invertebrate [89]. In fish, bacterial contamination is known to reduce sperm motility and fertility in multiple species, including Atlantic salmon (*Salmo salar*) [90], common carp (*Cyprinus carpio*) [91], and channel catfish (*Ictalurus punctatus*) [92]. Bacterial contamination in sperm suspensions can occur even when sterile techniques are used [93,94]. In amphibians, the Wolffian ducts empty into the cloaca [95], exposing spermic urine to internal (faecal material) and external (environmental) sources of bacteria within the cloaca, thus increasing the likelihood of bacterial contamination [79]. Reducing bacterial proliferation may be essential to improving sperm longevity during storage [54]. One way to combat bacteria is through the use of antibiotic treatment.

The addition of antibiotics to control bacteria in sperm samples is routinely used in humans, livestock, and aquaculture species [96,97]. In fish, penicillin, streptomycin, erythromycin, gentamicin, and neomycin are the most commonly used antibiotics [72,77]. The addition of antibiotics to fish semen has been reported to improve sperm quality (motility, velocity and viability) and longevity during short-term storage in species including common carp (*C. carpio*) [91], Atlantic cod (*Gadus morhua*), haddock (*Melanogrammus aeglefinus*) [98], Atlantic salmon (*S. salar*) [90], African catfish (*Clarias gariepinus*) [92], Caspian brown trout (*Salmo trutta caspius*) [99], Nile tilapia (*Oreochromis niloticus*) [96], spotted halibut (*Verasper variegatus*) [77], and other commercially valuable fish species [72]. For example, Rahimi et al. [99] observed that sperm motility was higher in Caspian brown trout (*S. trutta caspius*) sperm samples stored in storage medium with antibiotics added compared to the control group after three days of storage. Similarly, Zidni et al. [77] found that the addition of antibiotics resulted in improved sperm motility and viability in spotted halibut (*V. variegatus*), with sperm successfully stored for 60 days with antibiotic treatment compared to only 30 days in untreated suspensions. Based on the beneficial effects across species, treatment of semen with antibiotics is considered to be a critical component of prolonging the viability of sperm for aquaculture [72].

To date, the effect of antibiotics on amphibian sperm during short-term storage has only been tested in six studies spanning five amphibian species; one toad, and four frog species (Table 1). The duration of storage reported in these studies spans from four to 46 days, with assessments occurring either daily or every two to four days (Table 1). These studies sourced sperm from testes macerates and/or spermic urine following hormone therapy. It has been proposed that the addition of antibiotics to spermic urine may be more beneficial than to testes macerates, given that spermic urine has a greater diversity and abundance of bacteria [63], though more studies are required to confirm this pattern. Both the type and dose of antibiotics used varies greatly, with gentamicin or a penicillin–streptomycin combination most commonly employed. In European common frogs (*Rana temporaria*), a greater range of antibiotics was tested, including lincomycin, enrofloxacin, gentamicin, and a combination of each, at concentrations ranging 0.015–4 mg/mL (see Table 1; [80]). In this study, adding gentamicin at concentrations ranging 0.03–0.4 mg/mL to spermic urine had the most beneficial effect, with motility maintained for at least 32 days compared to untreated samples where motility was only maintained for a maximum of 12 days [80].

There may be several explanations for the varied results reported following the addition of antibiotics to amphibian sperm suspensions during cold storage (Table 1). Firstly, decreases in sperm motility and longevity following the addition of antibiotics at high concentrations may be due to the antibiotics’ toxic effect on sperm by reducing mitochondrial function [96]. Importantly, antibiotic toxicity, particularly with gentamicin, is dose dependent, with adverse effects typically reported at 4 mg/mL gentamicin, compared with beneficial or no effect at lower concentrations (Table 1). In half of the studies presented in Table 1, the bacterial abundance in suspensions treated with antibiotics was also quantified; this is important to understand the efficacy of antibiotic treatment for controlling bacterial abundance, as well as the associated influences on sperm parameters. The addition of antibiotics at low concentrations to sperm suspensions has the potential to improve the longevity of amphibian sperm during short-term storage significantly, though more research is needed. Future studies should seek to test a range of antibiotic concentrations on sperm performance in more species and also measure bacterial proliferation throughout storage to determine optimal concentrations that inhibit bacterial abundance without compromising sperm functionality, which may be species specific [63]. Irrespective of whether sperm viability is improved or unchanged by adding antibiotics, this treatment could have additional benefits, particularly when transporting samples between facilities. For biosecurity purposes, eliminating the transfer of bacteria and disease between captive facilities is of utmost importance [63].

Another important area for further research is investigating differences in bacterial abundance, species diversity, and effectiveness of antibiotic treatment, between spermic urine samples collected from wild versus captive individuals. There is evidence that captivity does affect skin, gut, and cloacal microbiomes in various vertebrates; however, no study to date has explored whether captivity affects the bacterial abundance or diversity of amphibian spermic urine samples. Wild-caught individuals of the Malaysian Mahseer fish (*Tor tambroides*) contain more diverse gut bacterial communities than captive counterparts [100]. Similarly, in several bird species, cloacal bacterial diversity has been shown to be lower in captive individuals compared with wild birds [101]. Similar results have also been demonstrated in amphibian species, with captive individuals having comparatively lower cutaneous bacterial diversity than their wild counterparts [102,103,104,105]. Whilst no studies to date have quantified the variation in cloacal bacterial communities of amphibians moved between wild and captive settings, it is predicted that wild amphibians would exhibit greater bacterial abundance and diversity. There is currently a greater emphasis on the collection of spermic urine samples from wild amphibians in order to facilitate genetic exchange between wild and captive populations, as well as to safeguard valuable genetic material from remnant wild populations with GRBs [10]. The risk of disease transmission and cross-infection from unwanted bacteria and infectious agents present within GRBs requires consideration [106]. For this reason, strict biosafety practices need to be developed and adhered to for all GRBs for the collection, storage, management and use of sperm suspensions (e.g., [107]). As such, investigating the bacterial communities of spermic urine obtained from wild and captive males and the effects of additional antibiotic treatments on the bacterial load of sperm suspensions, as well as sperm viability and longevity, will be valuable avenues for future research.

**Table 1:** Summary of the effects of antibiotics applied to amphibian sperm during short-term storage.

Species	Storage Duration	Antibiotic	Dose	Effects (Compared to a Control Group)	Sperm Source	Reference
*Anaxyrus fowleri* (Fowler’s toad)	4 days (assessed daily)	Penicillin- streptomycin	600 mU	No change in MOT; ↓ VIA; *n* = 15.	SU	[79]
*Crinia signifera* (Common eastern froglet)	12 days (assessed every 3 days)	Gentamicin	1 mg/mL	↑ VIA; BA reduced; n = 10.	SU	Silla, Anastas, and Byrne (unpub-lished data)
		Penicillin- streptomycin	1000 IU penicillin + 1 mg/mL streptomycin	↑ VIA; BA reduced; n = 10.	SU	
*Litoria booroolongensis* (Booroolong frog)	21 days (assessed every 3 days)	Gentamicin	4 mg/mL	↓ MOT; n = 10.	TM	[54]
	24 days (assessed every 4 days)	Gentamicin	1 mg/mL	No change in MOT; BA reduced; n = 8.	TM	[63]
			2 mg/mL	No change in MOT; BA near eliminated; n = 8.	TM	
			3 mg/mL	↓ MOT; BA near eliminated; n = 8.	TM	
			4 mg/mL	↓ MOT; BA near eliminated; n = 8.	TM	
	6 days (assessed daily)	Gentamicin	1 mg/mL	No change in MOT; BA reduced; n = 10.	SU	[63]
			2 mg/mL	No change in MOT; BA reduced; n = 10.	SU	
			3 mg/mL	No change in MOT; BA reduced; n = 10.	SU	
			4 mg/mL	No change in MOT; BA reduced; n = 10.	SU	
*Litoria ewingii* (Southern brown tree frog)	14 days (assessed every 2 days)	Gentamicin	2 mg/mL	No change in VIA; BA reduced; n = 10.	TM	[108]
			4 mg/mL	No change in VIA; BA reduced; n = 10.		
*Rana temporaria* (European common frog)	36 days (assessed every 3 days)	Lincomycin	0.4 mg/mL	No change in MOT; n = 5.	SU	[80]
		Enrofloxacin	1.2 mg/mL	↑ MOT; n = 5.	SU	
		Lincomycin-enrofloxacin-gentamicin	0.11–0.73–0.73 (total 1.5) mg/mL	↑ MOT; n = 5.	SU	
	46 days (assessed every 3–4 days)	Gentamicin	0.015 mg/mL	No change in MOT; n = 5.	SU	
			0.03 mg/mL	↑ MOT; no change in FERT; n = 5.	SU	
			0.06 mg/mL	↑ MOT; no change in FERT; n = 5.	SU	
			0.1 mg/mL	↑ MOT (>80% for 30 days); no change in FERT; n = 5.	SU	
			0.2 mg/mL	↑ MOT; n = 5.	SU	
			0.4 mg/mL	↑ MOT; n = 5.	SU	
			1 mg/mL	No change in MOT; n = 5.	SU	
			2 mg/mL	No change in MOT; n = 5.	SU	
			4 mg/mL	↓ MOT; n = 5.	SU	

MOT = motility; VIA = viability; FERT = fertilisation (as % to first cleavage); TM = testes macerates; SU = spermic urine; BA = bacterial abundance. Arrows indicate a significant increase or decrease compared to a control group that weren’t supplemented with antibiotics. Sample sizes are the number of replicates per treatment.

### 4.4. The Use of Antioxidants

The decline in sperm motility over time may, in part, be due to the accumulation of reactive oxygen species (ROS), which causes oxidative stress [109,110]. Whilst ROS is a natural by-product of cellular metabolism, oxidative stress can cause molecular damage, cellular dysfunction, and death [111]. Moreover, oxygen free radicals can reduce intracellular ATP levels, which can, in turn, reduce sperm cell functioning [112]. Sperm cells are particularly vulnerable to excess ROS production because they naturally produce large amounts of ROS and have low antioxidant capacity [113]. The damage caused by ROS can be inhibited by antioxidants, which are reducing enzymes that prevent the oxidation of ROS and thereby limit the cellular damage caused by oxidative stress [114]. Therefore, antioxidant supplementation in storage mediums may minimise ROS-associated cell damage to improve the short-term storage of sperm [115,116].

In mammals, the supplementation during short-term sperm storage with a range of antioxidants has been shown to be beneficial in several studies [109,117,118,119]. For example, in boar (*Sus scrofa*), several antioxidants including vitamin E and butylated hydroxytoluene (BHT) improve sperm parameters over time, compared to control suspensions without antioxidant supplementation [120]. In externally fertilising fish, the use of antioxidant supplementation during short-term storage has been investigated in several species, with promising results. For example, Lahnsteiner and Mansour [121] tested the effect of five antioxidants in storage media on sperm motility and viability at various concentrations after three and five days of storage in four fish species: burbot (*Lota lota*), perch (*Perca fluviatilis*), bleak (*Alburnus alburnus*), and brown trout (*S. trutta*). Of the five antioxidants tested, uric acid improved sperm motility and viability retention in all four species, with optimal concentrations ranging 0.5–1 mM for burbot (*L. lota*), perch (*P. fluviatilis*), and bleak (*A. alburnus*), and 0.25–0.5 mM for brown trout (*S. trutta*). Additionally, catalase improved sperm motility and viability in three of the four species, with optimal concentrations 2000 U/L for burbot (*L. lota*), 1000–2000 U/L for perch (*P. fluviatilis*) and 100 U/L for brown trout (*S. trutta*), and oxidised methionine improved sperm viability in all species when the concentration ranged 1.5–3 mM. Catalase is a key enzyme involved in neutralising hydrogen peroxide, a harmful oxidant [111], and methionine is a naturally occurring proteins that scavenges ROS [122]. Carnitine improved sperm motility and viability in bleak (*A. alburnus*) in concentrations 0.5–2 mM, whilst higher concentrations reduced sperm motility in perch (*P. fluviatilis*) and brown trout (*S. trutta*). Reduced glutathione at 3 mM improved sperm motility in perch (*P. fluviatilis*), and the oxidised form of glutathione at the same concentration improved sperm motility in bleak (*A. alburnus*)*;* however, the addition of glutathione resulted in no difference in sperm motility in burbot (*L. lota*) and brown trout (*S. trutta*) [121]. This study identified uric acid as the main antioxidant component of the seminal plasma in these teleost species and recommended supplementing the storage medium with uric acid at a concentration of 0.5 mM, as well as supplementation with methionine and catalase for some species. Another study in brown trout (*S. trutta*) found vitamin C to reduce sperm motility and viability during short term storage, though positive effects were found with the addition of methionine and uric acid [116].

In the rainbow trout (*Oncorhynchus mykiss*), the addition of antioxidants, specifically vitamin C, vitamin E, and polyphenol, improved sperm motility during short-term storage and led to improved fertilisation capacity of the sperm [123]. Similarly, in three salmonid species, Atlantic salmon (*S. salar*), coho salmon (*Oncorhynchus kisutch*), and rainbow trout (*O. mykiss*), storing sperm in medium supplemented with another antioxidant, sodium alginate, improved sperm motility and viability during ten days of storage [124]. Furthermore, in another study in coho salmon (*O. kisutch*), BHT, an antioxidant with additional antiviral and antibiotic properties, was supplemented in the semen at a concentration of either 1 or 2 mM, with a concentration of 2 mM found to significantly improve sperm motility after three days of storage [125]. Additionally, supplementing the storage medium of spotted halibut (*V. variegatus*) with the antioxidants mito-TEMPO, trehalose and glutathione improved sperm motility and viability during storage [77]. Overall, these studies conclude that supplementing the storage medium with antioxidants is a way to improve the storage of fish sperm by minimising oxidative stress.

Several antioxidant compounds, such as vitamin C, vitamin E, glutathione, and uric acid have been identified in the seminal plasma of fish [116,121,126]. To the best of our knowledge, the presence of antioxidant compounds in amphibian seminal plasma are yet to be identified. It is possible that sperm expelled within spermic urine are exposed to some level of natural antioxidant protection. However, levels of ROS have been shown to significantly increase with storage time in several fish species, and subsequent increases in the activity of naturally-occurring antioxidants are insufficient to counteract the negative effects [127,128]. As such, despite the presence of naturally-occurring antioxidants, fish semen benefits from additional antioxidant supplementation during storage [116,123,124,129], and the same may be true for amphibians.

In amphibians, the supplementation of storage mediums with antioxidants during the short-term storage of sperm is yet to be tested. However, two studies in the Booroolong frog (*L. booroolongensis*) tested the addition of antioxidants post-storage, by adding these components to the activation media. Specifically, the addition of caffeine and theophylline significantly increased sperm longevity and improved sperm motility eight, nine, and ten hours after activation, which was suggested to be due to the antioxidant properties of these compounds [130]. By comparison, the second study tested the effect of vitamin C and E, and found vitamin C to be detrimental to sperm motility across all treatment concentrations (0–0.25 µg/µL), and it was suggested that vitamin C might have become a pro-oxidant; that is, it may have produced oxygen byproducts. Vitamin E had no effect across all tested concentrations (0–1.75 µg/mL), and this may have been due to the already high endogenous antioxidant capacity in the sperm of this species [74]. It is currently unknown whether the addition of antioxidants may prove beneficial during short-term storage in amphibians, as has been reported for some fish species. Accordingly, future research should examine the effect of adding antioxidants such as glutathione or BHT to the storage medium on sperm parameters following short-term storage. Furthermore, there would be a benefit in quantifying ROS levels in sperm suspensions during storage by conducting lipid peroxidation tests [116], as the level of oxidative damage incurred during storage remains unknown in amphibians and may prove to be species-specific. High levels of ROS in sperm suspensions during storage might indicate that supplementation with antioxidants is beneficial.

In addition to supplementation during short-term storage, antioxidants might improve sperm cryopreservation outcomes by counteracting the increase in ROS during the cryopreservation process [120]. A review of 47 studies pertaining to the cryopreservation of boar (*S. scrofa*) sperm reported that most studies found the supplementation of cryopreservation media with antioxidants was beneficial for sperm fertilisation success [120]. Such benefits might also extend to other vertebrates. In humans, adding leptin improved sperm DNA quality [131], and in a study in boar, adding vitamin E improved sperm motility and viability [132]. Whilst the metabolism of mammalian sperm is different to that of external fertilisers, the beneficial effects of antioxidants have also been reported in externally fertilising fish species. In the Russian sturgeon (*Acipenser gueldenstaedti*), the addition of vitamin C to the cryopreservation medium not only improved sperm motility post-thaw, but also enhanced fertilisation capacity and reduced the level of chromosomal aberrations in the embryos fertilised with cryopreserved sperm [133]. A study on the common carp (*C. carpio*), reported increased post-thaw motility and longevity, in addition to increased fertilisation and hatching rates in sperm suspensions supplemented with cysteine [134]. Another study in Russian sturgeon (*A. gueldenstaedti*) found that supplementation with BHT improved sperm motility post-thaw [135]. Future studies in cryopreservation should consider whether adding antioxidants to the cryopreservation medium can improve post-thaw outcomes in amphibians, as this is yet to be tested.

## 5. Protocols for the Cryopreservation of Amphibian Sperm

Sperm cryopreservation offers additional benefits beyond those of short-term sperm storage, as these protocols allow for living genetic material to be stored in GRBs and used well into the future [19,136], extending the genetic lifespan of valuable individuals [137]. However, cryopreservation is technical and presents challenges to the living cell; the process involves dilution in cryopreservation medium and cooling to ultra-low temperatures, and each step can negatively affect sperm functional capacity [138]. Therefore, optimisation to minimise adverse effects is imperative, and studies in this space have been conducted in a wide range of species [138,139,140]. Protocol optimisation and standardisation is even more critical concerning endangered species, in which case threats to biodiversity may be imminent, making the need more pressing, and biological material may be limited [136]. In cases such as this, optimisation can occur in more abundant species within the same taxonomic family (e.g., [23]).

The earliest attempts to freeze and thaw sperm date back an astonishing ~250 years, with seminal experiments by Lazzaro Spallanzani in 1776 [141]. Amphibian sperm has been cryopreserved from as early as 1938, where the addition of sucrose resulted in 40% sperm motility following immersion in liquid nitrogen for ten seconds [142], and further work in 1946 and 1952 revealed that glycerol is an effective cryoprotectant, prolonging sperm survival during storage at −4 °C for days to weeks [143,144]. Following these early attempts, further research into amphibian sperm cryopreservation was largely lacking until the early nineties, when the recognition of the extent of global amphibian declines renewed interest in the field. In recent decades, amphibian sperm cryopreservation protocols have been developed in a variety of species, with research in this field following an upward trajectory. Until recently, sperm cryopreservation protocols were primarily developed using sperm obtained from testes macerates; however, this protocol is generally discouraged in threatened species due to the need to sacrifice males [15,145]. Of note, testes macerates obtained from common species can provide a source of concentrated high-quality sperm for optimisation experiments. Additionally, where an unexpected death occurs within a colony of threatened species, or a planned euthanasia occurs, testes macerates are a valuable resource that should be collected and cryopreserved for future application. In recent years, the successful cryopreservation of sperm obtained non-invasively as spermic urine or milt has been achieved in a growing number of threatened species, including the Chinese giant salamander (*Andrias davidianus*) [146], the Wyoming toad (*A. baxteri*) [147], the Houston toad (*Anaxyrus houstonensis*) [148], a Harlequin frog (*Atelopus* sp.) [149], the Panamanian golden frog (*Atelopus zeteki*) [150], the Hellbender (*C. alleganiensis*) [37], the Chiricahua leopard frog (*Lithobates chiricahensis*) [148], the dusky gopher frog (*L. sevosa*) [35,147], the green and golden bell frog (*L. aurea*)(R. Upton, unpublished data), the Booroolong frog (*L. booroolongensis*) (R. J. Hobbs et al., unpublished data), the yellow-spotted bell frog (*Litoria castanea*) (R. J. Hobbs and J. K. O’Brien, unpublished data), Littlejohn’s tree frog (*Litoria littlejohni*) (R. Upton, unpublished data), the black-spotted newt (*Notophthalmus meridionalis*) [151], the Puerto Rican crested toad (*P. lemur*) [148,152], Baw Baw frog (*Philoria frosti*) (A. J. Silla and R. J. Hobbs et al., unpublished data), the Southern corroboree frog (*P. corroboree*), the Northern corroboree frog (*Pseudophryne pengilleyi*) (R. J. Hobbs and J. K. O’Brien, unpublished data), and the Kweichow newt (*Tylototriton kweichowensis*) [151]. The cryopreservation of spermic urine does present some challenges. The primary issue is that the concentration of sperm, and often also the volume of sample obtained, is markedly lower in spermic urine than within sperm suspensions derived from testes macerates, which is further reduced when the samples are diluted in cryopreservation media pre-freeze and activation media post-thaw [64].

The process of cryopreservation exerts stress on sperm, and can minimise both functionality and survival [153]. The freezing process exposes sperm cells to osmotic stress, intracellular ice-crystal formation, and cryoprotectant toxicity, which can damage cells and make them unviable [154,155]. Thus, the cryoprotectant components within the cryopreservation medium and the cooling rate must be carefully considered and tested to develop successful cryopreservation protocols [156]. In addition, upon thawing, surviving sperm may exhibit damaged DNA, altered mRNA expression and epigenetic changes [157]. Additives to the cryopreservation medium and appropriate thawing methods can help minimise this damage, so optimisation of this is also necessary [155,157].

### 5.1. Cryopreservation Medium

The cryopreservation medium is essential in minimising adverse effects on sperm cells during the freeze-thaw process. Cryopreservation mediums may contain several different components and typically include penetrative cryoprotectant agents (CPAs; e.g., dimethyl formamide, glycerol), which act to reduce intracellular ice-formation combined with or without a non-penetrative CPA (e.g., sucrose, trehalose), appropriate concentrations of which help draw water from the cell along the osmotic gradient, protecting from solution effects such as excessive dehydration [158]. In addition, mediums may include antioxidants, buffers, membrane stabilisers, metabolic substrates, and/or antibiotics [136]. These media generally need to be formulated on a species-specific basis to account for physiological differences, such as surface to volume ratio of sperm cells, membrane permeability, and cold sensitivity [136,140,159,160]. In addition to deciding which components comprise the medium, the concentration of each component and time of exposure of samples to cryoprotectants prior to freezing may have species-specific effects [15,161].

Cryoprotectant agents can be classified into two categories: penetrating and non-penetrating [157]. Penetrating CPAs include dimethyl sulfoxide (DMSO), dimethylformamide (DMF, also referred to as DMFA), glycerol, dimethyl acetaldehyde, propylene glycol, ethylene glycol, and methanol [15,157,162,163]. These work by lowering the solution’s freezing point so that intracellular water can more slowly leave the cell before freezing and minimising the risk of intracellular ice formation [157]. However, these can be toxic at high concentrations [157], leading to reduced efficacy. Non-penetrating CPAs are compounds that are highly hydrated and non-ionic, including glucose, sucrose, and trehalose [158], and work by increasing extracellular osmolality to increase cell dehydration and preventing osmotic swelling during thawing [164].

In amphibians, published reports documenting the effect of a variety of CPAs at different concentrations, with the addition of different extenders, spans fifty species across ten families and two orders (Table 2). Bufonidae and Pelodryadidae are the most represented of these families, with 11 and 17 species spanning 15 and five studies, respectively. These studies sourced sperm from testes macerates and spermic urine/milt following hormonal induction of spermiation; of the species studied, 23 sourced sperm from testes macerates alone, 25 utilised spermic urine/milt alone, and two utilised both testes macerates and spermic urine, across multiple studies (Table 2). Additionally, in one species, the axolotl (*A. mexicanum*) sperm was sourced by collecting spermatophores deposited by live males [165,166]. The base medium (e.g., SAR, motility inhibiting solution (MIS), and suspension buffer (SB)), the type of CPA (both penetrating and non-penetrating), the addition of extra components, and the concentration of each component varied among studies. However, the most commonly used penetrating CPAs were DMF and DMSO at final concentrations between 5–15%; the most commonly used non-penetrating CPAs were sucrose and trehalose, with final concentration most commonly at 10%; and the additives in some CPA solutions were either egg yolk, bovine serum albumin (BSA), or foetal bovine serum (FBS) at concentrations ranging from 2.5% to 50% (Table 2). Whilst most studies measured absolute sperm motility (total % motile) as a sperm quality parameter, others also included sperm viability (%; also referred to as membrane integrity), the fertility rate (% eggs to first cleavage), the gastrulation rate (% embryos to reach gastrulation), and hatching rate (% embryos hatched); three only measured relative motility (% motile sperm following cryopreservation relative to % motile sperm before cryopreservation) [53,167,168].

The motility, viability, fertility, and hatching rates of frozen–thawed sperm varied greatly among treatments and species. When the CPA treatment included either DMF or DMSO, post-thaw sperm motility (absolute) spanned 25–58% in studies utilising testes macerates and 15–74% in studies utilising spermic urine, and sperm viability spanned 24–60% and 30–72% for studies utilising testes macerates and spermic urine, respectively. Thawed spermic urine samples showed high recovery of sperm motility in several species when the cryoprotectant was either DMF or DMSO; however, overall, results vary considerably (see Table 2). Whilst sperm motility and viability are almost always significantly higher in fresh compared to frozen–thawed sperm, successful fertilisation, embryonic and larval development, development to sexual maturity, and generation of an F1 generation using frozen–thawed sperm have been shown to be possible in several species (see Section 6 below).

Across the studies outlined in Table 2, other factors beyond the CPAs chosen may have influenced the resulting sperm parameters, including other components of the cryopreservation medium, such as proteins or other supplements, the sperm packaging materials, and freezing and thawing rates. This variety in methods makes it difficult to draw conclusions regarding the influence of cryopreservation and specific components of the process on sperm viability and performance; however, some explanations should be considered. Regarding CPA concentration, a balance between the concentration being high enough to sufficiently protect sperm cells from cryoinjury and low enough to be non-toxic to sperm must be met [169]. In amphibians, a final concentration of 5–15% DMSO or DMF may provide this balance; however, susceptibility to CPA toxicity is species specific [35], which may provide an explanation for the differences in optimal concentrations among and within species. Furthermore, the addition of other components, including BSA and FBS (e.g., [38,55,56,60,148,151,168,170]), may further reduce adverse effects on sperm cells by acting as antioxidants, increasing membrane fluidity and further dehydrating the sperm cells [38], and so may explain the higher sperm parameters reported in some cases. Future studies should seek to test protocol transferability between model and endangered species, continue to test differing types and concentrations of CPAs in a wider range of amphibian species, and test whether the inclusion of additional components into the cryopreservation medium can improve post-thaw sperm outcomes.

**Table 2:** Summary of cryoprotectants used for amphibian sperm cryopreservation, where sperm parameters (motility, viability, or fertilisation rates) were assessed.

Family	Species	Conservation Status (IUCN)	Cryoprotectants	Sperm Parameters Post-Thaw (%)	Sperm Source	Reference
Ambystomatidae	*Ambystoma mexicanum * (Axolotl) ^	CR	10% SC	53 (VIA); n = 4	SP	[166]
			6% DMA	58 (VIA); n = 9	SP *	[165]
	*Ambystoma tigrinum* (Eastern tiger salamander) ^	LC	0.5% BSA	0 (MOT); n = 10	SM	[171]
			5% TRE	0 (MOT); n = 10	SM	
			5% SC	7 (MOT), 2 (FPM); n = 10	SM	
			5% DMSO	6 (MOT), 1 (FPM); n = 10	SM	
			5% DMSO + 5% TRE	14 (MOT), 4 (FPM); n = 10	SM	
			5% DMSO + 5% SC	6 (MOT), 1 (FPM); n = 10	SM	
			5% DMSO + 0.5% BSA	10 (MOT), 8 (FPM); n = 10	SM	
Bufonidae	*Anaxyrus americanus* (American toad)	LC	15% EG	88 (relative MOT); n = 6	TM	[168]
			20% EG	66 (relative MOT); n = 6	TM	
			20% EG + 75% FBS	98 (relative MOT); n = 2	TM	
			20% DMSO	77 (relative MOT); n = 3	TM	
		LC	4% DMSO + 50% FBS	34 (VIA); n = 6	TM	[55]
	*Anaxyrus baxteri* (Wyoming toad)	EW	5% DMF + 10% TRE	22 (relative MOT); n = 14	SU	[147]
	*Anaxyrus boreas boreas* (Mountain boreal toad)	LC	10% DMF + 10% TRE	49 (MOT), 23 (FPM); n = 6	SU	[35]
			5% DMF + 10% TRE	43 (MOT), 28 (FPM); n = 6	SU	
			10% DMSO + 10% TRE	49 (MOT), 28 (FPM); n = 6	SU	
			5% DMSO + 10% TRE	43 (MOT), 23 (FPM); n = 6	SU	
	*Anaxyrus fowleri* (Fowler’s toad)	LC	10% DMF + 10% TRE	69 (MOT), 39 (FPM); n = 6	SU	[35]
			5% DMF + 10% TRE	63 (MOT), 44 (FPM); n = 6	SU	
			10% DMSO + 10% TRE	69 (MOT), 42 (FPM); n = 6	SU	
			5% DMSO + 10% TRE	57 (MOT), 35 (FPM); n = 6	SU	
			5% DMF + 10% TRE + 0.25% BSA	26 (MOT), 19 (FERT); n = 32	SU	[38]
			10% DMF + 10% TRE + 0.25% BSA	30 (MOT), 15 (FERT); n = 32	SU	
			10% DMSO + 10% TRE + 0.25% BSA	27 (MOT), 14 (FERT); n = 32	SU	
			5% DMF + 10% TRE	30 (MOT), 8 (FPM), 30 (VIA), 16 (HAT); n = 20	SU	[147]
			10% DMF + 10% TRE	5 (MOT); n = 20	SU	
	*Anaxyrus houstonensis* (Houston toad)	CR	10% DMF + 10% TRE + 0.25% BSA	32 (relative MOT); n = 8	SU	[148]
	*Atelopus* sp. (undescribed Harlequin frog)	VU	5% DMF + 1% SC	50 (MOT), ~20 (FPM), ~50 (VIA); n = 4	SU	[149]
			5% DMF + 2.5% SC	~50 (MOT), ~27 (FPM), ~50 (VIA); n = 4	SU	
			5% DMF + 5% SC	~30 (MOT), ~8 (FPM), 54 (VIA); n = 4	SU	
			5% DMF + 1% TRE	~30 (MOT), ~2 (FPM), ~40 (VIA); n = 4	SU	
			5% DMF + 2.5% TRE	~30 (MOT), ~4 (FPM), ~40 (VIA); n = 4	SU	
			5% DMF + 5% TRE	~15 (MOT), ~1 (FPM), ~45 (VIA); n = 4	SU	
	*Atelopus zeteki* (Panamanian golden frog)	CR	5% DMF + 10% TRE + 10% EY	~5–10 (MOT); n = 4	SU	[150]
			10% DMF + 10% TRE + 10% EY	~5–10 (MOT); n = 4	SU	
	*Epidalea calamita* (Natterjack toad)	LC	10% DMF + 10% SC	50 (FERT), 37 (HAT); n = 16	SU	[172]
	*Peltophryne lemur* (Puerto Rican crested toad)	EN	10% DMF + 10% TRE + 0.25% BSA	32 (relative MOT); n = 12	SU	[148]
			10% DMF + 10% TRE	28 (MOT), 4 (FERT); n = 8, 5	SU	[152]
			10% DMSO + 10% TRE	25 (MOT); n = 7	SU	
			10% DMF + 10% TRE	51 (MOT), 38 (FPM); n = 6	SU	[35]
			5% DMF + 10% TRE	47 (MOT), 34 (FPM); n = 6	SU	
			10% DMSO + 10% TRE	45 (MOT), 28 (FPM); n = 6	SU	
			5% DMSO + 10% TRE	44 (MOT), 30 (FPM); n = 6	SU	
	*Rhaebo guttatus* (SSmooth-sided toad)	LC	5% DMF + 10% TRE	33 (MOT),17 (FPM); n = 2	SU	[173]
	*Rhinella marina* (Cane toad)	LC	5% DMSO	19 (GAST); n = 6	TM	[174]
			10% DMSO	23 (GAST); n = 6	TM	
			15% DMSO	8 (GAST); n = 6	TM	
			10% DMSO + 10% SC	25 (MOT), 33 (FERT); n = 4	TM	[175]
			15% DMSO + 10% SC	47 (MOT), 61 (FERT); n = 4	TM	
			20% DMSO + 10% SC	34 (MOT), 42 (FERT); n = 4	TM	
			10% GLY + 10% SC	12 (MOT), 34 (FERT); n = 4	TM	
			15% GLY + 10% SC	25 (MOT), 14 (FERT); n = 4	TM	
			20% GLY + 10% SC	42 (MOT), 81 (FERT); n = 4	TM	
			10% DMSO + 10% EY	14 (relative MOT); n = 3	TM	[167]
			15% DMSO + 10% EY	36 (relative MOT); n = 3	TM	
			20% DMSO + 10% EY	51 (relative MOT); n = 3	TM	
			10% GLY + 10% EY	33 (relative MOT); n = 3	TM	
			15% GLY + 10% EY	34 (relative MOT); n = 3	TM	
			20% GLY + 10% EY	30 (relative MOT); n = 3	TM	
Cryptobranchidae	*Andrias davidianus* (Chinese giant salamander)	CR	10% DMSO	15 (VIA); n = unspecified	SM	[146]
	*Cryptobranchus alleganiensis* (Hellbender)	VU	5% DMSO	80 (MOT); n = unspecified	SM	[37]
			2.5% DMSO	20 (MOT); n = 5	SM	[176]
Eleutherodactylidae	*Eleutherodactylus coqui* (Puerto Rican coqui)	LC	7% GLY + FBS	20 (VIA); n = 6	TM	[56]
			7% DMSO + FBS	28 (VIA); n = 6	TM	
			20% SC + FBS	30 (VIA); n = 6	TM	
Limnodynastidae	*Limnodynastes peronii* (Striped marsh frog)	LC	20% GLY + 10% SC	20 (relative MOT); n = unspecified	TM	[53]
	*Philoria frosti * (Baw Baw frog)	CR	10% DMF + 10% TRE	17 (MOT), 59 (VIA); n = 4	SU	Silla and Hobbs et al. (unpublished data)
Myobatrachidae	*Crinia signifera * (Common eastern froglet)	LC	15% DMSO + 10% SC	15 (relative MOT); n = unspecified	TM	[53]
	*Pseudophryne bibronii* Bbrown toadlet)	LC	15% DMSO + 10% SC	20 (relative MOT); n = unspecified	TM	[53]
	*Pseudophryne corroboree* (Southern corroboree frog)	CR	10% DMF + 10% TRE	66 (VIA); n = 1	SU	Hobbs and O’Brien (unpublished data)
	*Pseudophryne pengilleyi* (Northern corroboree frog)	CR	10% DMF + 10% TRE	8 (MOT), 58 (VIA); n = 1	TM	Hobbs and O’Brien (unpublished data)
			10% DMF + 10% TRE	81 (VIA); n = 1	SU	Hobbs and O’Brien (unpublished data)
Pelodryadidae	*Litoria aurea* (Green and golden bell frog)	VU	10% DMSO + 10% SC	~35 (FPM), ~60 (VIA); n = 5	TM	[36]
			15% DMSO + 10% SC	~42 (FPM), ~60 (VIA), 13 (FERT), 2 (HAT); n = 5, 2	TM	
			20% DMSO + 10% SC	~18 (FPM), ~65 (VIA); n = 5	TM	
			10% GLY + 10% SC	~15 (FPM), ~40 (VIA); n = 5	TM	
			15% GLY + 10% SC	~8 (FPM), ~50 (VIA), ~10 (FERT), 0 (HAT); n = 5, 2	TM	
			20% GLY + 10% SC	~3 (FPM), ~45 (VIA); n = 5	TM	
			15% DMSO + 1% SC	~53 (MOT), ~53 (VIA); n = 4	SU	Upton et al. (unpublished data)
			15% DMSO + 10% SC	~14 (MOT), ~30 (VIA); n = 4	SU	
	*Litoria booroolongensis* (Booroolong frog)	CR	10% DMF + 10% TRE	~62 (MOT), ~60 (VIA); n = 8	SU	Hobbs et al. (unpublished data)
			15% DMSO + 1% SC	~60 (MOT), ~58 (VIA); n = 8	SU	
	*Litoria brevipalmata* (Green-thighed frog)	EN	15% DMSO + 10% SC	58 (MOT); n = unspecified	TM	[53]
			15% GLY + 10% SC	28 (MOT); n = unspecified	TM	
	*Litoria castanea* (Yellow-spotted bell frog)	CR	10% DMF + 10% TRE	66 (MOT), 24 (FPM), 56 (VIA); n = 3	SU	Hobbs and O’Brien (unpublished data)
	*Litoria citropa* (Blue Mountains tree frog)	LC	15% DMSO + 1% SC	~22 (FPM), ~62 (VIA); n = 3	TM	[23]
			15% DMSO + 10% SC	~5 (FPM), ~53 (VIA); n = 3	TM	
	*Litoria dentata* (Bleating tree frog)	LC	15% DMSO + 10% SC	45 (relative MOT); n = unspecified	TM	[53]
	*Litoria fallax* (Eastern dwarf tree frog)	LC	15% DMSO + 10% SC	85 (relative MOT); n = unspecified	TM	[53]
			10% DMSO + 1% SC	~30 (FPM), ~50 (VIA); n = 4	TM	[23]
			10%DMSO + 10% SC	~18 (FPM), ~52 (VIA); n = 4	TM	
			15% DMSO + 1% SC	~55 (FPM), ~73 (VIA); n = 4	TM	
			15% DMSO + 10% SC	~10 (FPM), ~62 (VIA); n = 4	TM	
	*Litoria latopalmata* (Broad-palmed frog)	LC	15% DMSO + 10% SC	70 (relative MOT); n = unspecified	TM	[53]
	*Litoria littlejohni* (Littlejohn’s tree frog)	LC	15% DMSO + 1% SC	~58 (MOT), ~71 (VIA); n = 3	SU	Upton et al. (unpublished data)
	*Litoria lesueurii* (Lesueur’s tree frog)	LC	15% DMSO + 10% SC	100 (relative MOT); n = unspecified	TM	[53]
			15% GLY + 10% SC	100 (relative MOT); n = unspecified	TM	
			15% DMSO + 1% SC	~35 (MOT), ~65 (VIA); n = 3	TM	[23]
			15% DMSO + 10% SC	~20 (MOT), ~54 (VIA); n = 3	TM	
	*Litoria nasuta* (Striped rocket frog)	LC	15% DMSO + 10% SC	80 (relative MOT); n = unspecified	TM	[53]
	*Litoria nudidigitus * (Southern leaf green tree frog)	LC	15% DMSO + 1% SC	~45 (MOT), ~52 (VIA); n = 3	TM	[23]
			15% DMSO + 10% SC	~22 (MOT), ~41 (VIA); n = 3	TM	
	*Litoria peronii* (Peron’s tree frog)	LC	15% DMSO + 10% SC	90 (relative MOT); n = unspecified	TM	[53]
			20% GLY + 10% SC	81 (relative MOT); n = unspecified	TM	
	*Litoria phyllochroa* (Leaf green tree frog)	LC	15% DMSO + 10% SC	78 (relative MOT); n = unspecified	TM	[53]
	*Litoria quiritatus * (Screaming tree frog)	NL	10% DMSO + 1% SC	~25 (FPM), ~58 (VIA); n = 4	TM	[23]
			10%DMSO + 10% SC	~5 (FPM), ~32 (VIA); n = 4	TM	
			15% DMSO + 1% SC	~65 (FPM), 83 (VIA); n = 4	TM	
			15% DMSO + 10% SC	~2 (FPM), ~43 (VIA); n = 4	TM	
	*Litoria subglandulosa* (New England tree frog)	VU	15% DMSO + 10% SC	60 (relative MOT); n = unspecified	TM	[53]
	*Litoria tyleri* (Tyler’s tree frog)	LC	15% DMSO + 1% SC	~55 (FPM), ~83 (VIA); n = 3	TM	[23]
			15% DMSO + 10% SC	~20 (FPM), ~75 (VIA); n = 3	TM	
Pipidae	*Xenopus laevis* (African clawed frog)	LC	14% SC + 0.08% NAHCO_3_ + 20% EY	10 (VIA); n = 4	TM	[177]
			14% SC + 0.08% NAHCO_3_ + 20% EY	22 (FERT); n = 10	TM	[178]
			5% DMSO	53 (MOT), 38 (VIA), 29 (HAT); n = 3	TM	[58]
			10% DMSO	49 (MOT), 35 (VIA), 9 (HAT); n = 3	TM	
			5% DMSO + 1% SC	62 (MOT), 43 (VIA), 28 (HAT); n = 3	TM	
			5% DMSO + 2.5% SC	71 (MOT), 60 (VIA), 48 (HAT); n = 3	TM	
			5% DMSO + 5% SC	60 (MOT), 35 (VIA), 27 (HAT); n = 3	TM	
			27% SC + 1.7% NAHCO_3_ + 20% EY	40 (FERT); n = 1	TM	[59]
			15% DMSO + 10% SC/4% DMSO + 50% FBS	25–50% (MOT); 0 (FERT); n = unspecified	TM	[170]
	*Xenopus tropicalis* (Western clawed frog)	LC	14% SC + 0.08% NAHCO_3_ + 20% EY	40 (VIA); n = 4	TM	[177]
			14% SC + 0.08% NAHCO_3_ + 20% EY	44 (FERT); n = 9	TM	[178]
			27% SC + 1.7% NAHCO_3_ + 20% EY	57 (FERT); n = 2	TM	[59]
Ranidae	*Lithobates catesbeianus* (American Bullfrog)	LC	1–7.5% DMSO	0 (MOT); n = unspecified	SU	[179]
			1–7.5% methanol	0 (MOT); n = unspecified	SU	
	*Lithobates chiricahuensis* (Chiricahua leopard frog)	VU	10% DMF + 10% TRE + 0.25% BSA	74 (relative MOT); n = 10	SU	[148]
	*Lithobates pipiens* (Northern leopard frog)	LC	4% DMSO + 50% FBS	55 (VIA); n = 5	TM	[55]
			5% GLY + 12% EY	71 (VIA); n = 10	SU	[180]
			15% DMSO + 10% SC	44 (VIA); n = 10	SU	
			5% DMF + 10% TRE	58 (relative MOT), 13 (HAT); n = 17	SU	[147]
			10% DMF + 10% TRE	4 (relative MOT); n = 17	SU	
	*Lithobates sevosus* (Dusky gopher frog)	CR	5% DMF + 10% TRE	38 (relative MOT); n = 13	SU	[147]
			10% DMF + 10% TRE	2 (relative MOT); n = 13	SU	
			5% DMF + 10% TRE	73 (MOT), 32 (FPM), 55 (FERT); n = 5	SU	[35]
	*Lithobates sylvaticus* (Wood frog)	LC	4% DMSO	31 (VIA); n = 5	TM	[55]
			2% methanol	15 (VIA); n = 5	TM	
			4% DMSO + 50% FBS	43 (VIA); n = 5	TM	
			2% methanol + 50% FBS	35 (VIA); n = 5	TM	
			14% GLY	13 (VIA); n = 5	TM	[181]
			28% GLY	17 (VIA); n = 5	TM	
			12% DMSO	10 (VIA); n = 5	TM	
			23% DMSO	13 (VIA); n = 5	TM	
			2% GC	23 (MOT), 45 (VIA); n = 3	TM	[62]
			1% GLY	18 (MOT),45 (VIA); n = 3	TM	
	*Pelophylax lessonae* (Pool frog)	LC	24% DMF + 20% SC	40 (MOT), 10 (FPM), 71 (VIA), 29 (FERT); n = 7	SU	[182]
	*Rana temporaria * (European common frog)	LC	15% DMSO + 10% TRE + 0.5% BSA	47 (MOT), 41 (VIA), 53 (FERT); n = 10	TM	[60]
			15% DMF + 10% TRE + 0.5% BSA	58 (MOT), 50 (VIA), 68 (FERT); n = 10	TM	
			5% GLY	15 (MOT), 11 (VIA), 2 (HAT); n = 3	TM	[61]
			10% DMSO	33 (MOT), 32 (VIA), 14 (HAT); n = 3	TM	
			5% GLY + 2.5% SC	45 (MOT), 34 (VIA); n = 3	TM	
			10% DMSO + 2.5% SC	37 (MOT), 43 (VIA), 3 (HAT); n = 3	TM	
			5% GLY + 2.5% SC + 5% EY	44 (MOT), 60 (VIA), 12 (HAT); n = 3	TM	
			10% DMSO + 2.5% SC + 5% EY	29 (MOT), 30 (VIA), 5 (HAT); n = 3	TM	
			12% DMSO + 10% SC	43 (MOT), 46 (VIA), 81 (FERT), 54 (HAT); n = 3	SU	[183]
			12% DMSO + 10% SC + 10% EY	36 (MOT), 38 (VIA), 91 (FERT), 58 (HAT); n = 3	SU	
			12% DMF + 10% SC	65 (MOT), 72 (VIA), 91 (FERT), 71 (HAT); n = 3	SU	
Salamandridae	*Notophthalmus meridionalis* (Black-spotted newt) ^	EN	10% DMSO + 1% BSA	5 (MOT), 0 (FPM); n = 6	SM	[151]
			10% DMSO + 1% BSA + 10% TRE	6 (MOT), 0 (FPM); n = 6	SM	
	*Tylototriton kweichowensis* (Kweichow newt) ^	VU	10% DMSO + 1% BSA	14 (MOT), 0 (FPM); n = 4	SM	[151]
			10% DMSO + 1% BSA + 10% TRE	12 (MOT), 0 (FPM); n = 4	SM	

Conservation Status: LC = least concern; VU = vulnerable; EN = endangered; CR = critically endangered; EW = extinct in the wild; NL = not listed. Cryoprotectants: DMF = Dimethyl formamide (also referred to in the literature as DMFA); DMSO = Dimethyl sulfoxide; DMA = Dimethyl acetamide; EG = ethylene glycol; GLY = glycerol; TRE = trehalose; SC = sucrose; GC = glucose; FBS = foetal bovine serum; BSA = bovine serum albumin; EY = egg yolk; cryoprotectant percentages are final concentration following dilution. Findings: MOT = total motility (%); relative MOT = post-thaw motility relative to pre-freeze motility (%); FPM = forward progressive motility (%); VIA = viability (%); FERT = fertilisation (% to first cleavage); GAST = gastrulation (% embryos reaching gastrulation); HAT = hatchlings (% embryos hatched). Sperm source: TM = testes macerates; SU = spermic urine; SM = spermic milt; SP = spermatophores; SP * = sperm cells extracted from spermatophore. ^ denotes species which exhibit internal fertilisation. Sample sizes are the number of replicates per treatment.

### 5.2. Freezing and Thawing Methods

Alongside the cryopreservation medium, both the cooling and thawing rates impact the possibility of cryoinjury, and optimum rates need to be investigated to minimise damaging effects [184]. Combined cooling and freezing rates (referred to from hereon as cooling rates) can be divided into two categories: slow-cooling, (temperature change < −200 °C/min); or ultra-rapid cooling (temperature change > −1000 °C/min), which is generally coupled with the process of vitrification [35,185,186]. Vitrification, where both the cooling rate and the concentration of CPAs is very high, aims to prevent the formation of ice crystals by directly converting components into a glassy state [186,187]. For this review, the focus will be on slow-cooling methods, the most commonly employed type of freezing in amphibian sperm cryopreservation in recent decades, as vitrification attempts in amphibian species are associated with low to no recovery of viable amphibian sperm [175]. Within the slow-cooling category exists differences in the optimal rate and method of freezing, owing to high levels of species-specificity in tolerance to cryoinjury [162,188], furthered by the diversity in reproductive modes seen in amphibians [95].

In amphibians, different cooling rates are generally applied via one of three methods. Firstly, programmable freezers can be used to alter cooling rates and achieve step-wise freezing; secondly, samples can be held in liquid nitrogen vapour at differing heights above the surface of the liquid nitrogen; and thirdly, samples can be suspended at different heights within a charged dry shipper. Cooling via either approach is generally followed by direct transfer into liquid nitrogen upon reaching at least −80 °C [189] or lower (Table 3). Regarding thawing, the rate at which samples are thawed is generally reciprocal to the rate of freezing; the faster the cooling, the faster the thawing rate required [190]. Optimisation of thawing rate may be required to minimise, or prevent, the recrystallisation of intra- and extra-cellular ice during warming [191]. Commonly, slow-cooled samples are thawed either by immersion in a warm water bath (20–40 °C) or thawing in air at room temperature [64,192].

Studies in amphibian sperm cryopreservation have used various freezing and thawing methods (Table 3). Whilst both the freezing and thawing methods varied across studies, the most common method is freezing in liquid nitrogen vapour and thawing in a water bath (20–40 °C) (Table 3). Commonly used methods of thawing included thawing in air, or a combination of exposure to air and a water bath (Table 3). Of the 20 studies which held samples in liquid nitrogen vapour, 11 held samples 5 to 20 centimetres above the surface of liquid nitrogen for 5 to 15 min; six studies held samples at varied distances above liquid nitrogen for 7 to 15 min and; and three studies held samples at unspecified distances above liquid nitrogen (Table 3). Several studies additionally measured the freeze rate achieved following these methods (Table 3). It is to be noted that the type of sample packaging can impact sample cooling rates; the majority of studies packed samples within cryogenic straws; however, several studies used Eppendorf tubes, cryovials, or unspecified units with the capacity ranging 0.05–1.5 mL. In all studies, controlled cooling was followed by plunging samples into liquid nitrogen. In studies which utilised programmable freezing machines, one had a constant cooling rate of around −10 °C/min [59], and five used stepped cooling ramps [23,36,53,57,175], which involved a ten-minute acclimation at 2 °C followed by cooling rates of −1, 3, and 3.4/5 °C/min until the samples reached −80 °C, at which point samples were submerged in liquid nitrogen. 

Studies that compared multiple cooling rates provide valuable insight into optimal cooling rates across species. Most protocols had frozen sperm at a slower rate (<−30 °C/min) (Table 3), with Naranjo et al. (2021) [149] demonstrating significantly higher post-thaw sperm parameters when samples were held 10 and 13 cm above liquid nitrogen (57% motility and 59% viability, and 60% motility and 58% viability, respectively), as opposed to 5 and 7 cm above liquid nitrogen (17% motility and 16% viability, and 29% motility and 18% viability, respectively) in an undescribed species of harlequin frog (*Atelopus* sp.); however, cooling rates were not quantified. Similar results were obtained by Mansour [61] in European common frogs (*R. temporaria*), where samples held at 10 cm above liquid nitrogen resulted in 30–35% post-thaw sperm motility, whereas samples held at 5 cm above liquid nitrogen were completely immotile. These results indicate that slower freezing is optimal, which could be due to faster freezing rates causing damage to plasma membranes [149], or disintegration of the sperm body or the flagella [175]. Conversely, other studies demonstrated improved sperm motility following freezing at faster rates (>−30 °C/min), with Burger et al. [38] freezing sperm 5 cm above liquid nitrogen (−32 to −45 °C/min) resulting in significantly higher sperm motility compared to freezing at 10 cm above liquid nitrogen (−20 to −29 °C/min) in Fowler’s toad (*A. fowleri*). A follow-up study [148] also found that freezing 5 cm above liquid nitrogen resulted in significantly higher post-thaw sperm motility in two additional species: the Puerto Rican crested toad (*P. lemur*) and the Chiricahua leopard frog (*L. chiricahuensis*). In addition, this method also resulted in higher sperm motility in the Houston toad (*A. houstonensis*), though this result was not statistically significant [148]. The variation in post-thaw sperm parameters between freezing rates is difficult to explain due to large differences in freezing methods, including the composition of the cryopreservation medium, the packaging used, and the source of sperm (spermic urine or testes macerates). Additionally, differences in post-thaw sperm quality are likely to be influenced by inter-specific differences in susceptibility of sperm to cryoinjury.

Whilst optimal cooling rates generally appear to be <−30 °C/min, this has been shown to vary on a species-specific basis. Future studies should aim to continue to test and compare a range of freezing rates across a wider range of amphibian species, as cooling rate has been shown to significantly affect post-thaw sperm outcomes. Furthermore, future studies should aim to study cooling rates in a wider diversity of endangered species, especially using spermic urine, to test protocol transferability between taxonomic groups.

## 6. Generating Offspring from Cryopreserved Sperm

The generation of offspring derived from AF using frozen–thawed sperm (referred to from hereon as ‘cryo-derived offspring’) has been reported in a range of non-amphibian vertebrate taxa, including mammals [193], birds [194,195], and fish [196,197]. Whilst offspring have been successfully generated using cryopreserved sperm in a range of species, including in livestock and humans, there are few examples where offspring performance has been monitored throughout development, or in subsequent generations. However, a study in mice reported that offspring generated from frozen–thawed sperm that had been cryopreserved for more than ten years were successfully able to reproduce and were not phenotypically different from mice within the standard breeding colony [198]. In the case of humans, long-term follow-up studies in children generated using cryopreserved sperm have reported no differences in development or rate of abnormalities, though these studies have rarely extended beyond birth [199,200]. Additionally, care must be taken when interpreting results from human studies, as the assessed cohort typically have prior fertility issues, and comparisons are typically made with the general populace rather than with other children produced using AI with fresh sperm (i.e., to allow comparisons with the same cohort presenting to fertility clinics). This has led to a limited understanding of the long-term effects of cryopreservation on offspring performance and viability. In terms of conservation, the quality of offspring, in addition to the quantity of offspring produced, may be critical in determining the success of CBPs and subsequent reintroductions [201]. It is therefore necessary to investigate whether the viability of cryo-derived individuals differs from offspring generated from AF using fresh sperm as well as offspring from natural amplexus. More importantly, we need to gain an understanding of what impact, if any, differences have on the long-term fitness of populations. Of note, if cryo-derived offspring are found to differ from naturally-generated offspring, this does not discount the value of cryo-derived offspring to conservation but will highlight a need for further protocol refinement to reduce such effects, in addition to research into ‘compensatory’ practices (such as enhanced tadpole nutrition to encourage compensatory growth of smaller individuals).

Offspring performance following AF with cryopreserved sperm has been assessed in a variety of fish species, with initial results suggesting variable fitness effects. Studies in a number of species, including the olive barb (*Puntius sarana*) [202], silver barb (*Barbonymus gonionotus*) [203], Tiete tetra (*Brycon insignis*) [204], and tilapia (*Oreochromis* sp.) [205] have reported that cryo-derived offspring performed similarly to offspring produced from AF using fresh sperm. By contrast, studies in other species, including brown trout (*S. trutta*) [206], rainbow trout (*O. mykiss*) [207], small-scaled pacu (*Piaractus mesopotamicus*) [208], wels catfish (*Siluris glanis*) [209], and yellow perch (*Perca falvenscens*) [210] have found that several fitness-determining traits displayed shifts that might compromise fitness. For example, in brown trout (*S. trutta*), though there was no difference in fertilisation rates, growth, or survival up to the embryo stage; larval growth was significantly reduced after hatching in cryo-derived offspring [206]. Nusbaumer et al. [206] suggested two possible explanations for this finding: first, as there is often phenotypic variation among sperm within an ejaculate, the process of cryopreservation may select for certain types of sperm cells; second, they suggested that cryopreservation causes cryoinjury to the sperm, which may affect cellular integrity, DNA integrity, and the sperm epigenome, which can, in turn, impact offspring. In mice, fertilisation of oocytes by intracytoplasmic sperm injection (ICSI) with DNA-damaged sperm caused by a suboptimal cryopreservation protocol resulted in premature ageing and altered behaviour in offspring [211], though use of optimally cryopreserved sperm for AF and embryo transfer did not appear to be detrimental to offspring development or adult viability [198]. In addition, a study in a freshwater fish species, the Tambaqui (*Colossoma macropomum*), reported that frozen–thawed sperm had decreased DNA methylation, which is a key mechanism for epigenetic regulation [212]. This decrease was suggested as a possible explanation for low numbers of viable embryos and hatchlings following AF [212]. With the goal of directing new conservation approaches, there is an increased need to explore the effects that cryopreservation may have on offspring fitness in a range of species, especially those of conservation concern.

To date, there have been a limited number of studies assessing the fitness-determining traits (FDTs) of amphibian cryo-derived offspring. Since 2011, a total of 12 amphibian studies, involving eight species, have been conducted which generated cryo-derived offspring (Table 4). In these studies, sperm suspensions were cryopreserved using various methods (see Table 2 and Table 3) and, after thawing, were used in AF trials to fertilise fresh oocytes to generate and assess cryo-derived offspring. AF was conducted by expelling the thawed contents of cryogenic straws/vials directly onto fresh oocytes placed in Petri dishes immediately after oocyte deposition (Table 4). Following a 5–30 min period of dry fertilisation, dishes were flooded with ether distilled water or SAR. Oocytes were collected by stripping (manual abdominal massage) following hormonal induction of ovulation [35,38,152,154,178,213,214], collected from amplectant females after first signs of oviposition [36,57] or collected directly from the oviducts following euthanasia [183]. Prior to AF, sperm were reactivated in either distilled water [36,57,213,214], sterilised embryo transfer water [38,145,152,154], Marc’s modified Ringer [178], or saline solution. In addition, Upton et al. [57] centrifuged sperm prior to reactivation and AF to wash samples of CPAs. These studies measured various fitness-determining traits across various life stages, with large variation among studies in which specific fitness-determining traits were assessed and the outcomes reported (Table 4). 

### 6.1. Hatching Success and Larval Survival

In the amphibian studies that have compared hatching success between cryo-derived offspring and offspring generated from AF using fresh sperm, the use of frozen–thawed sperm for AF generally appears to result in lower fertilisation and hatching success ([34,35,38,145,152,154,183]; Table 4). However, the magnitude of this difference varies across species, with significant differences only demonstrated in four of the eight species studied (Table 4). Interestingly, there was also one species where a positive correlation was reported. In a study of Eastern dwarf tree frogs (*Litoria fallax*), Upton et al. [57] reported that hatching success was significantly higher in cryo-derived offspring. However, sub-optimal AF conditions, such as the osmolality of the fertilisation medium, were thought to have generated this outcome [57]. Despite this variation in findings, each of the aforementioned studies produced hatchlings from cryopreserved sperm (Table 4). Beyond hatching success, studies in four species have assessed larval survival. A study by Shishova et al. [183] conducted in European common frogs (*R. temporaria*) measured larval survival seven days post AF and found the percentage of surviving larvae to be similar to that of the control group (offspring generated from AF using fresh sperm). Similarly, there was no difference in larval survival to metamorphosis between groups in Fowler’s toad (*A. fowleri*) [213], Puerto Rican crested toads (*P. lemur*) [152], and Eastern dwarf tree frogs (*L. fallax*) [57] (Table 4). Importantly, hatching success and larval survival, among other traits, can also be influenced by maternal and paternal effects and genetic incompatibilities (see discussion below); therefore, future studies should aim to conduct full-diallel cross breeding designs to control for these effects.

### 6.2. Morphological Traits

Morphological trait variation can impact fitness in a variety of ways in amphibians. For example, larger body size at metamorphosis has been linked to increased fecundity [215,216], increased mating success [217], and superior foraging ability [218,219,220]. Morphological trait variation can also significantly affect population viability [213]. Furthermore, the effects of smaller body size at early life stages can have long-term effects that influence viability in later life stages [221]. Of the limited number of amphibian studies that have quantified aspects of larval and juvenile morphology, results have been equivocal. One study reported no difference in mass at metamorphosis between treatment cryo-derived offspring and those that were generated via AF using fresh sperm in African clawed frogs (*Xenopus laevis*) [178]. Two studies report larger size in cryo-derived offspring. Specifically, Langhorne [35] found no difference in snout-vent length between treatment groups in the critically endangered dusky gopher frog (*L. sevosus*), but cryo-derived metamorphs were significantly heavier [35]. Additionally, in Eastern tiger salamanders (*Ambystoma tigrinum*), larval length was significantly longer in cryo-derived offspring at 28 days post-hatching [145]. In contrast, a study on Fowler’s toad (*A. fowleri*) found that larvae were significantly smaller in size (measured as total length, tail length, and larval body width) in cryo-derived offspring compared to offspring from natural amplexus [213]. Additionally, in a separate study, two measures of larval morphology (total length and tail length) were significantly smaller in cryo-derived offspring in the same species [214]. Furthermore, Poo et al. [213] and Poo and Hinkson [214] found that both mass and snout-vent length at metamorphosis were significantly lower in cryo-derived offspring. Of note, these studies in Fowler’s toad did not utilise a full-diallel cross-breeding design (whereby the male sperm suspension/ejaculates and female clutch are split and crossed in every pairwise combination), so individual maternal and paternal effects or genetic incompatibilities between particular dam/sire combinations were unaccounted for, which may have influenced the results reported. There is a need for more controlled experimental work aimed at elucidating general patterns in the type of traits influenced, and the magnitude of the effects. Critically, whilst past studies may have split clutches or ejaculates across some experimental treatments, no amphibian study to date has employed a full-diallel cross-breeding design. Moving forward, studies would benefit from using a full-diallel cross split-clutch/split-ejaculate breeding design to control for maternal and paternal effects as well as sire/dam incompatibilities as these effects can strongly influence amphibian fitness [13]. By using this approach, we will be better positioned to disentangle offspring phenotypic differences from parental effects compared to effects linked to the cryopreservation process. Importantly, if future research utilising full split-clutch/split-ejaculate mating designs find reduced offspring size in cryo-derived offspring, it will be important for subsequent research to investigate if the smaller size of tadpoles or metamorphs can be countered by compensatory growth, which may be achieved by enhancing captive diet and nutrition prior to reintroduction [222]. It will also be important to quantify the long-term impacts of any differences in offspring morphology on the fitness of populations post-reintroduction.

### 6.3. Survival to Sexual Maturity

Several studies spanning a variety of amphibian species have demonstrated that cryo-derived offspring can successfully reach sexual maturity (see Table 4). Additionally, a small number of studies aimed to test the reproductive viability of cryo-derived offspring, through the production of an F2 generation (Table 4). The findings reported in these studies were variable, though encouraging, as cryo-derived offspring were used to successfully generate offspring in each case. In a recent report by Lampert et al. [154], an F1 generation of three species of amphibians, the Eastern tiger salamander (*A. tigrinum*), the Puerto Rican crested toad (*P. lemur*), and the dusky gopher frog (*L. sevosus*), were produced via AF using frozen–thawed sperm and fresh oocytes. These offspring were then reared to sexual maturity and used to produce an F2 generation (using one cryo-derived F1 female with a control male for the Eastern tiger salamander and one cryo-derived F1 male with a control female for both the Puerto Rican crested toad and the dusky gopher frog). In each case, an F2 generation was successfully produced. The reproductive viability of cryo-derived offspring has previously been demonstrated in Puerto Rican crested toads (*P. lemur*) [152] and dusky gopher frogs (*L. sevosa*) [34], and has additionally been demonstrated in African clawed frogs (*X. laevis*) [178]. Furthermore, the ability for green and golden bell frogs (*L. aurea*) [36] and Eastern dwarf tree frogs (*L. fallax*) [57] to reach sexual maturity suggests the production of an F2 generation is also possible in these species.

Moving forward, it would be valuable for future studies to assess a range of offspring-fitness-determining traits, with an emphasis on assessing the reproductive viability of cryo-derived offspring and determining if there are long-term transgenerational effects of cryopreservation [154]. Such work will provide key insights into the most effective pathways for integrating cryopreservation into CBPs.

## 7. Conclusions

Reproductive technologies have the potential to aid in amphibian threatened species recovery. Specifically, advances in cold storage and cryopreservation of amphibian sperm, coupled with assisted fertilisation technologies, are valuable tools for long-term genetic management and are being applied to an increasing number of threatened species globally. Protocols have been developed in both the short-term cold storage and long-term cryopreservation of amphibian sperm in a variety of species, which can be used to facilitate genetic exchange between captive and wild populations, as well as to expand the reproductive lifespan of individuals. Recent advances have enabled the generation of amphibian offspring from frozen–thawed sperm in a growing variety of species. Continued research is now required to determine whether cryo-derived offspring and their successive generations vary morphologically or behaviourally from naturally bred counterparts, which will be important to direct future conservation actions. Such work will provide key insights into the applicability and scope of reproductive technologies, including cryopreservation, to safeguard amphibian diversity globally.

## Figures and Tables

**Table 3:** Summary of freezing and thawing protocols for amphibian sperm cryopreservation. Summary of sperm parameters (motility, viability) are included for studies where multiple cooling rates were tested.

Family	Species	Conserva-tion Status (IUCN)	Cooling Rate (−° min^−1^)	Freeze Method	Thaw Method	Sperm Parameters Post-Thaw (%)	Sperm Source	Reference
Ambystomatidae	*Ambystoma mexicanum* (Axolotl)	CR	~108	Within goblet in dry shipper	WB (25 °C; 5 min)	85 (VIA)	SP	[166]
			~24	Within goblet and canister in dry shipper	WB (25 °C; 5 min)	86 (VIA)	SP	
			~10	Within additional insulation in dry shipper	WB (25 °C; 5 min)	89 (VIA)	SP	
			NS	5 cm above LN for 15 min	15 °C; 5 min		SP *	[165]
	*Ambystoma tigrinum* (Eastern tiger salamander)	LC	NS	20 cm above LN for 15 min	WB (40 °C; ~5 s)		SM	[171]
Bufonidae	*Anaxyrus americanus* (American toad)	LC	0.7	Direct into −20 °C freezer; store in −80 °C freezer	WB (25 °C; 10 s)		TM	[168]
			130	Direct into −80 °C freezer	WB (NS)		TM	[55]
	*Anaxyrus baxteri* (Wyoming toad)	EW	~10	Above LN	Air (23 °C; 10 s); WB (40 °C; 10 s)		SU	[147]
	*Anaxyrus boreas boreas* (Mountain boreal toad)	LC	20–25	10 cm above LN	WB (40 °C; ~5 s)		SU	[35]
	*Anaxyrus fowleri* (Fowler’s toad)	LC	32–45	5 cm above LN for 10 min	WB (40 °C; ~5 s)	24 (MOT), 5 (FPM)	SU	[38]
			20–29	10 cm above LN for 10 min	WB (40 °C; ~5 s)	19 (MOT), 4 (FPM)	SU	
			20–25	10 cm above LN	WB (40 °C; ~5 s)		SU	[35]
			~9	Above LN2.	Air (23 °C; 10 s); WB (40 °C; 10 s)		SU	[147]
	*Anaxyrus houstonensis* (Houston toad)	CR	32–45	5 cm above LN for 10 min	WB (40 °C; ~5 s)	32 (relative MOT)	SU	[148]
			20–29	10 cm above LN for 10 min	WB (40 °C; ~5 s)	27 (relative MOT)	SU	
	*Atelopus* sp. (undescribed Harlequin frog)	VU	NS	5 cm above LN for 10 min	WB (40 °C; 15 s)	17 (MOT), 0 (FPM), 16 (VIA)	SU	[149]
			NS	7 cm above LN for 10 min	WB (40 °C; 15 s)	29 (MOT), 1 (FPM), 18 (VIA)	SU	
			NS	10 cm above LN for 10 min	WB (40 °C; 15 s)	57 (MOT), 24 (FPM), 59 (VIA)	SU	
			NS	13 cm above LN for 10 min	WB (40 °C; 15 s)	60 (MOT), 22 (FPM), 58 (VIA)	SU	
	*Atelopus zeteki* (Panamanian golden frog)	CR	60	10 cm above LN for 3 min	WB (21 °C; 15 s)		SU	[150]
	*Epidalea calamita* (Natterjack toad)	LC	NS	5 cm above LN for 10 min	WB (40 °C; ~5 s)		SU	[172]
	*Peltophryne lemur* (Puerto Rican crested toad)	EN	32–45	5 cm above LN for 10 min	WB (40 °C; ~5 s)	32 (relative MOT)	SU	[148]
			20–29	10 cm above LN for 10 min	WB (40 °C; ~5 s)	27 (relative MOT)	SU	
			38	10 cm above LN for 10 min	WB (40 °C; ~5 s)		SU	[152]
			20–25	1 cm above LN	WB (40 °C; ~5 s)		SU	[35]
	*Rhaebo guttatus* (Smooth-sided toad)	LC	~10	10 cm above LN for 10 min	Air (23 °C; 10 s); WB (40 °C; 10 s)		SU	[173]
	*Rhinella marina* (Cane toad)	LC	1–5	Programmable freezer #	Air (21 °C)	47 (MOT)	TM	[175]
			~40 C	1 cm above LN	Air (21 °C)	<2% (MOT)	TM	
			~30	3 cm above LN	Air (21 °C)	<10% (MOT)	TM	
			1–5	Programmable freezer #	Air (21 °C)		TM	[167]
			NS	Direct into −20 °C freezer; store in −80 °C freezer	Air (4 °C) for 2 h		TM	[174]
Cryptobranchidae	*Andrias davidianus* (Chinese giant salamander)	CR	NS	Above LN	WB (26–28 °C)		SM	[146]
	*Cryptobranchus alleganiensis* (Hellbender)	VU	NS	5–10 cm above LN for 5 min	Rubbed between hands for several mins		SM	[37]
			NS	Direct into −80 °C freezer	WB (35 °C; ~10 s)		SM	[176]
Eleutherodactylidae	*Eleutherodactylus coqui* (Puerto Rican coqui)	LC	~24	Direct into −80 °C freezer	WB (20 °C; ~36 s)		TM	Michael and Jones [56]
Limnodynastidae	*Limnodynastes peronii* (Striped marsh frog)	LC	1–5	Programmable freezer #	Air		TM	[53]
	*Philoria frosti* (Baw Baw frog)	CR	~21	Within goblet and canister in dry shipper, 2-step lowering of goblet	Air (19–22 °C; 2 s); WB (40 °C; 5 s)		SU	Hobbs and O’Brien (unpublished data)
Myobatrachidae	*Crinia signifera* (Common eastern froglet)	LC	1–5	Programmable freezer #	Air		TM	[53]
	*Pseudophryne bibronii* (Brown toadlet)	LC	1–5	Programmable freezer #	Air		TM	[53]
	*Pseudophryne corroboree* (Southern corroboree frog)	CR	~21	Within goblet and canister in dry shipper, 2-step lowering of goblet	Air (19–22 °C; 2 s); WB (40 °C; 5 s)		SU	Hobbs and O’Brien (unpublished data)
	*Pseudophryne pengilleyi* (Northern corroboree frog)	CR	~21	Within goblet and canister in dry shipper, 2-step lowering of goblet	Air (19–22 °C; 2 s); WB (40 °C; 5 s)		SU	Hobbs and O’Brien (unpublished data)
Pelodryadidae	*Litoria aurea* (Green and golden bell frog)	VU	1–3.4	Programmable freezer #	Air (21 °C; 2 min)		TM	[36]
			1–3.4	Programmable freezer #	Air (21 °C; 2 min)		SU	Upton et al. (unpublished data)
	*Litoria booroolongensis* (Booroolong frog)	CR	~21	Within goblet and canister in dry shipper, 2-step lowering of goblet	Air (19 °C; 2 s); WB (40 °C; 5 s)		SU	Hobbs and O’Brien (unpublished data)
	*Litoria brevipalmata* (Green-thighed frog)	EN	1–5	Programmable freezer #	Air		TM	[53]
	*Litoria castanea* (Yellow-spotted bell frog)		~21	Within goblet and canister in dry shipper, two-step lowering of goblet	Air (19 °C; 2 s); WB (40 °C; 5 s)		SU	Hobbs and O’Brien (unpublished data)
	*Litoria citropa* (Blue Mountains tree frog)	LC	1–3.4	Programmable freezer #	Air (21 °C; 2 min)		TM	[23]
	*Litoria dentata* (Bleating tree frog)	LC	1–5	Programmable freezer #	Air		TM	[53]
	*Litoria fallax* (Eastern dwarf tree frog)	LC	1–5	Programmable freezer #	Air		TM	[53]
			1–3.4	Programmable freezer #	Air		TM	[23,57]
	*Litoria latopalmata* (Broad-palmed frog)	LC	1–5	Programmable freezer #	Air		TM	[53]
	*Litoria lesueurii* (Lesueur’s tree frog)	LC	1–5	Programmable freezer #	Air		TM	[53]
	*Litoria littlejohni* (Littlejohn’s tree frog)	LC	1–3.4	Programmable freezer #	Air (21 °C; 2 min)		SU	[23]
	*Litoria nasuta* (Striped rocket frog)	LC	1–5	Programmable freezer #	Air		TM	[53]
	*Litoria nudidigitus* (Southern leaf green tree frog)	LC	1–3.4	Programmable freezer #	Air (21 °C; 2 min)		TM	[23]
	*Litoria peronii* (Peron’s tree frog)	LC	1–5	Programmable freezer #	Air		TM	[53]
	*Litoria phyllochroa* (Leaf green tree frog)	LC	1–5	Programmable freezer #	Air		TM	[53]
	*Litoria quiritatus* (Screaming tree frog)	NL	1–3.4	Programmable freezer #	Air (21 °C; 2 min)		TM	[23]
	*Litoria subglandulosa* (New England tree frog)	VU	1–5	Programmable freezer #	Air		TM	[53]
	*Litoria tyleri* (Tyler’s tree frog)	LC	1–3.4	Programmable freezer #	Air (21 °C; 2 min)		TM	[23]
Pipidae	*Xenopus laevis* (African clawed frog)	LC	NS	Direct into −80 °C freezer	Held between hands		TM	[177]
			NS	Direct into −80 °C freezer	WB (37 °C; 40 s)		TM	[178]
			NS	5 cm above LN for 7 min	Air (40 s)	1 (MOT), 6 (VIA)	TM	[58]
			NS	8 cm above LN for 7 min	Air (40 s)	8 (MOT), 15 (VIA)	TM	
			20–25	10 cm above LN for 7 min	Air (40 s)	20 (MOT), 48 (VIA)	TM	
			10	Programmable freezer	Air (40 s)		TM	[59]
			NS	Direct into −80 °C freezer	WB (37 °C)		TM	[170]
	*Xenopus tropicalis* (Western clawed frog)	LC	NS	Direct into −80 °C freezer	Held between hands		TM	[177]
			NS	Direct into −80 °C freezer	WB (37 °C; 40 s)		TM	[178]
			10	Programmable freezer	Air (40 s)		TM	[59]
Ranidae	*Lithobates catesbeianus* (American bullfrog)	LC	NS	Dry shipper	WB (37 °C; 10 s)		SU	[179]
	*Lithobates chiricahuensis* (Chiricahua leopard frog)	VU	32–45	5 cm above LN for 10 min	WB (40 °C; ~5 s)	74 (relative MOT)	SU	[148]
			20–29	10 cm above LN for 10 min	WB (40 °C; ~5 s)	55 (relative MOT)	SU	
	*Lithobates pipiens* (Northern leopard frog)	LC	130	Direct into −80 °C freezer	WB (NS)		TM	[55]
			NS	On dry ice for 3 min.	Centrifuge (21 °C)		SU	[180]
			~11	Above LN	Air (23 °C; 10 s); WB (40 °C; 10 s)		SU	[147]
	*Lithobates sevosus* (Dusky gopher frog)	CR	~12	Above LN	Air (23 °C; 10 s); WB (40 °C; 10 s)		SU	[147]
			20–25	10 cm above LN	WB (40 °C; ~5 s)		SU	[35]
	*Lithobates sylvaticus* (Wood frog)	LC	~130	Direct into −80 °C freezer	WB		TM	[55]
			~130	On dry ice; stored in −80 °C freezer	WB (30 °C)		TM	[181]
			~0.2	Direct into −8 °C freezer	WB (23 °C for 20 min)		TM	[62]
	*Pelophylax lessonae* (Pool frog)	LC	NS	10 cm above LN for 15 min	WB (40 °C)		SU	[182]
	*Rana temporaria* (European common frog)	LC	5–7	Above LN for 13 min	WB (40 °C; 6–8 s)		TM	[60]
			NS	5 cm above LN for 7 min	WB (22 °C; 30–40 s)	0 (MOT)	TM	[61]
			NS	10 cm above LN for 7 min	WB (22 °C; 30–40 s)	30–35 (MOT)	TM	
			5–7	10 cm above LN for 5–7 min	WB (40 °C)		SU	[183]
Salamandridae	*Notophthalmus meridionalis* (Black-spotted newt)	EN	NS	10 cm above LN for 10 min	WB (40 °C; 5 s)		SM	[151]
	*Tylototriton kweichowensis* (Kweichow newt)	VU	NS	10 cm above LN for 10 min	WB (40 °C; 5 s)		SM	[151]

Conservation Status: LC = least concern; VU = vulnerable; EN = endangered; CR = critically endangered; EW = extinct in the wild; NL = not listed. NS = not specified; LN = liquid nitrogen (held in vapour); WB = water bath. Findings: MOT = motility (%); relative MOT = post-thaw motility relative to pre-freeze motility (%); FPM = forward progressive motility (%); VIA = viability. Sperm source: TM = testes macerates; SU = spermic urine; SM = spermic milt; SP = spermatophores; SP * = sperm cells extracted from spermatophore. # denotes when programmable freezers utilised freeze ramps (not detailed here).

**Table 4:** Summary of studies that produced offspring using cryopreserved amphibian sperm, detailing fitness-determining traits (FDTs) examined and subsequent key results, with offspring produced from thawed cryopreserved sperm (cryo) compared to offspring produced from fresh/cold-stored sperm (non-cryo).

Family	Species	Conservation Status (IUCN)	Sperm Source	FDTs Assessed	Key Results	Reference
Ambystomatidae	*Ambystoma tigrinum* (Eastern tiger salamander)	LC	SM	1. Hatching success 2. Reproductive success of F1 generation	1. Cryo (33%) = non-cryo (45%); 21 cryo F1 hatchings produced 2. 1 cryo F1 female + 1 non-cryo male produced 5 F2 hatchlings (4% of eggs)	[154]
			SM	1. Larval total length 21 days post-hatching 2. Larval total length 28 days post-hatching 3. Survival beyond 28 days post-hatching	1. Cryo = non-cryo 2. Cryo > non-cryo 3. Cryo = non-cryo; continuation of development	[145]
Bufonidae	*Anaxyrus fowleri* (Fowler’s toad)	LC	SU	1. Hatching success	1. Cryo (13%) < non-cryo (52%); 1102 cryo F1 hatchlings produced Nb: hatchling progress currently being monitored	[38]
			SU	1. Larval total length 10 days post-oviposition 2. Larval tail length 10 days post-oviposition 3. Larval width 10 days post-oviposition 4. Larval total length 20 days post-oviposition 5. Larval tail length 20 days post-oviposition 6. Larval width 20 days post-oviposition 7. Larval duration 8. Survival to metamorphosis 9. Mass at metamorphosis 10. SVL at metamorphosis	1. Cryo = non-cryo 2. Cryo = non-cryo 3. Cryo = non-cryo 4. Cryo < non-cryo 5. Cryo < non-cryo 6. Cryo < non-cryo 7. Cryo > non-cryo 8. Cryo = non-cryo 9. Cryo < non-cryo 10. Cryo < non-cryo	[213]
			SU	1. Larval total length 30 days post-oviposition 2. Larval tail length 30 days post-oviposition 3. Larval width 30 days post-oviposition 4. Larval baseline activity level 5. Larval duration 6. Mass at metamorphosis 7. SVL at metamorphosis	1. Cryo < non-cryo 2. Cryo < non-cryo 3. Cryo = non-cryo 4. Cryo = non-cryo 5. Cryo = non-cryo 6. Cryo < non-cryo 7. Cryo < non-cryo	[214]
	*Peltophyrne lemur* (Puerto Rican crested toad)	EN	SU	1. Hatching success 2. Reproductive success of F1 generation	1. Cryo (15%) = non-cryo (27%); 46 cryo F1 hatchings produced 2. 1 cryo F1 male + 1 non-cryo female produced 5095 F2 hatchlings (98% of eggs)	[154]
			SU	1. Hatching success 2. Survival to metamorphosis 3. Survival of F1 generation 4. Reproductive success of F1 generation	1. Cryo (18%) = non-cryo (41%); 55 cryo F1 hatchlings produced 2. Cryo (15%) = non-cryo (27%); 46 cryo F1 metamorphs produced 3. 24 (75%) cryo F1 in CBP surviving after 1.5 years 4. 1 cryo F1 male produced F2 offspring	[152]
Pelodryadidae	*Litoria aurea* (Green and golden bell frog)	VU	TM	1. Embryonic duration 2. Hatching success 3. Survival to sexual maturity	1. Cryo = non-cryo 2. Cryo (~2%) < non-cryo (~40%) 3. Both male and female cryo F1 reached sexual maturity	[36]
	*Litoria fallax* (Eastern dwarf tree frog)	LC	TM	1. Hatching success 2. Survival to metamorphosis 3. Survival to sexual maturity	1. Cryo (62%) > non-cryo (30%); 133 cryo F1 hatchlings produced 2. Cryo = non-cryo; 4 cryo F1 metamorphs produced 3. 1 cryo F1 reached sexual maturity.	[57]
Pipidae	*Xenopus laevis* (African clawed frog)	LC	TM	1. Embryonic development 2. Mass at metamorphosis 3. Reproductive success of F1 generation	1. Cryo = non-cryo 2. Cryo = non-cryo 3. Cryo = non-cryo; 18 cryo F1 used for AF.	[178]
Ranidae	*Lithobates sevosus* (Dusky gopher frog)	VU	SU	1. Hatching success 2. Reproductive success of F1 generation	1. Cryo (4%) = non-cryo (7%); 42 cryo F1 hatchings produced 2. 1 cryo F1 male + 1 non-cryo female produced 48 F2 hatchlings (64% of eggs)	[154]
			SU	1. Hatching success 2. Reproductive success of F1 generation 3. Rate of development of F2 generation	1. Cryo (26%); non-cryo (46%) 2. 2 cryo F1 male + 1 non-cryo female produced F2 hatchlings 3. F2 = non-cryo	[34]
			SU	1. Hatching success 2. Mass at metamorphosis 3. SVL at metamorphosis 4. Survival to completion of metamorphosis 5. Rate of development	1. Cryo (27%) < non-cryo (46%) 2. Cryo > non-cryo 3. Cryo = non-cryo 4. Cryo (70%); non-cryo (55%) 5. Cryo = non-cryo	[35]
	*Rana temporaria* (European common frog)	LC	SU	1. Hatching success 2. Larval survival at 7 days post-fertilisation	1. Cryo (71%) = non-cryo 2. Cryo (74%) = non-cryo Nb: when cryo treatment was 12% DMF + 10% sucrose	[183]

Conservation Status: LC = least concern; VU = vulnerable; EN = endangered. Sperm source: TM = testes macerates; SU = spermic urine; SM = spermic milt. SVL = snout-vent length. Key results: cryo denotes offspring generated from AF using frosen-thawed sperm; < denotes significantly less than; > denotes significantly greater than; = denotes no significant difference; percentages for hatching success are % of cleaved embryos that successfully hatched.

## Data Availability

Data requests can be directed to the corresponding author.
